# Effect of altered production and storage of dopamine on development and behavior in *C. elegans*

**DOI:** 10.3389/ftox.2024.1374866

**Published:** 2024-08-16

**Authors:** Irene Lee, Ava C. Knickerbocker, Charlotte R. Depew, Elizabeth L. Martin, Jocelyn Dicent, Gary W. Miller, Meghan L. Bucher

**Affiliations:** ^1^ Department of Environmental Health Sciences, Mailman School of Public Health at Columbia University, New York, NY, United States; ^2^ Department of Molecular Pharmacology and Therapeutics, Vagelos College of Physicians and Surgeons, Columbia University, New York, New York, NY, United States

**Keywords:** dopamine, *Caenorhabditis elegans*, neurodevelopment, neurotoxicity, neuroscience

## Abstract

**Introduction:**

The nematode, *Caenorhabditis elegans* (*C. elegans*), is an advantageous model for studying developmental toxicology due to its well-defined developmental stages and homology to humans. It has been established that across species, dopaminergic neurons are highly vulnerable to neurotoxicant exposure, resulting in developmental neuronal dysfunction and age-induced degeneration. *C. elegans*, with genetic perturbations in dopamine system proteins, can provide insight into the mechanisms of dopaminergic neurotoxicants. In this study, we present a comprehensive analysis on the effect of gene mutations in dopamine-related proteins on body size, development, and behavior in *C. elegans.*

**Methods:**

We studied *C. elegans* that lack the ability to sequester dopamine (OK411) and that overproduce dopamine (UA57) and a novel strain (MBIA) generated by the genetic crossing of OK411 and UA57, which both lack the ability to sequester dopamine into vesicles and, additionally, endogenously overproduce dopamine. The MBIA strain was generated to address the hypothesis that an endogenous increase in the production of dopamine can rescue deficits caused by a lack of vesicular dopamine sequestration. These strains were analyzed for body size, developmental stage, reproduction, egg laying, motor behaviors, and neuronal health utilizing multiple methods.

**Results:**

Our results further implicate proper dopamine synthesis and sequestration in the regulation of *C. elegans* body size, development through larval stages into gravid adulthood, and motor functioning. Furthermore, our analyses demonstrate that body size in terms of length is distinct from the developmental stage as fully developed gravid adult *C. elegans* with disruptions in the dopamine system have decreased body lengths. Thus, body size should not be used as a proxy for the developmental stage when designing experiments.

**Discussion:**

Our results provide additional evidence that the dopamine system impacts the development, growth, and reproduction in *C. elegans*. Furthermore, our data suggest that endogenously increasing the production of dopamine mitigates deficits in *C. elegans* lacking the ability to package dopamine into synaptic vesicles. The novel strain, MBIA, and novel analyses of development and reproduction presented here can be utilized in developmental neurotoxicity experiments.

## Introduction

The model organism *Caenorhabditis elegans* (*C. elegans; “*worms”) has significant utility for developmental toxicology research due to its small size, transparent body, defined developmental staging, and eutelic nature (Brenner, 1974; Corsi et al., 2015). In comparison to the complex dopamine systems consisting of thousands of dopaminergic neurons in the human brain, *C. elegans* has a simple dopaminergic system consisting of eight neurons—six in the head and two in the body (White et al., 1986). Despite the difference in the complexity and number of neurons, dopamine regulates similar behaviors in *C. elegans* as in humans, including locomotion, food response, enhancement of odor avoidance, mating behavior, plasticity of the mechanosensory response, and behavioral choice (Sanyal et al., 2004; Kindt et al., 2007; Kimura et al., 2010; Correa et al., 2012; Omura et al., 2012; Wang et al., 2014).

In humans, dopamine synthesis occurs when tyrosine is converted to levodopa (L-DOPA) by tyrosine hydroxylase (TH) and L-DOPA is decarboxylated to dopamine by aromatic amino acid decarboxylase (Molinoff and Axelrod, 1971; Hare and Loer, 2004). Following synthesis, dopamine is sequestered into synaptic vesicles by vesicular monoamine transporter 2 (VMAT2), which is a necessary process for proper neurotransmission and to limit the amount of free dopamine in the cytosol that has the potential to be neurotoxic (Liu and Edwards, 1997). *C. elegans* contains homologs for human TH and VMAT2 encoded by the *cat-2* and *cat-1* genes, respectively (Duerr et al., 1999; Lints and Emmons, 1999). The VMAT2 homolog in *C. elegans*, *cat-1*, was reported to be 47% identical to human VMAT1 and 49% identical to human VMAT2, demonstrating the uptake of dopamine and serotonin (Erickson and Eiden, 1993; Erickson et al., 1996; Duerr et al., 1999). Furthermore, the uptake of these substrates was inhibited by known VMAT inhibitors tetrabenazine and reserpine, demonstrating an inhibitor profile similar to that of mammalian VMAT2 (Erickson and Eiden, 1993; Duerr et al., 1999).

Due to the high degree of conserved biology between *C. elegans* and humans, and the genetic tractability of *C. elegans*, transgenic strains with gene mutations in proteins involved in dopamine homeostasis can be easily generated to investigate human conditions. For instance, we have previously evaluated the effect of genetic- or toxicant-induced disruptions in the vesicular packaging of dopamine on neuronal health by comparing a genetic model of *C. elegans* (OK411) that lacks the neurotransmitter transporter necessary for packaging dopamine into synaptic vesicles and *C. elegans* exposed to the dopaminergic neurotoxicant 1-methyl-4-phenylpyridinium (MPP^+^) (Bradner et al., 2021). In *C. elegans*, *cat-1*-null animals demonstrate minor egg-laying deficits and decreased mating performance in male animals (Loer and Kenyon, 1993; Duerr et al., 1999). These animals also display age-dependent degeneration and increased susceptibility to the dopaminergic neurotoxicant and VMAT2 substrate MPP^+^ (Bradner et al., 2021). These findings substantiate those from previous work on rodent models, demonstrating that manipulating VMAT2 expression can alter the amount of dopamine in the cytosol; mediate neuronal vulnerability to degeneration, particularly in cases of toxicant exposures; and cause behavioral effects (Takahashi et al., 1997; Gainetdinov et al., 1998; Fumagalli et al., 1999; Staal and Sonsalla, 2000; Mooslehner et al., 2001; Caudle et al., 2007; Taylor et al., 2011; Hall et al., 2014; Bucher et al., 2020).

In Parkinson’s disease, there is significant evidence of dysfunctional VMAT2 expression and activity, suggesting the pathogenic role of dysregulated dopamine sequestration in disease onset and/or progression (Miller et al., 1999; Sala, Brighina et al., 2010; Piflt et al., 2014). The motor deficits resulting from deficient dopamine transmission in Parkinson’s disease are treated by supplementation with the precursor to dopamine, L-DOPA (Birkmayer and Hornykiewicz, 1961; Birkmayer and Hornykiewicz, 1962). In *C. elegans*, *cat-2* overexpression is used as an endogenous model of L-DOPA exposure to replicate an increase in dopamine production; however, these animals show age-dependent dopaminergic neurodegeneration (Manalo and Medina, 2018; Manalo and Medina, 2020). In the current study, we sought to characterize the effects of dysregulated dopamine production and storage on *C. elegans* development and determine whether endogenously increasing dopamine production through the overexpression of *cat*-*2* could rescue the deficits identified in *cat-1*-null *C. elegans.* To accomplish this, we bred two parent *C. elegans* strains OK411 (*cat-1* ^*null*^) and UA57 (*cat-2* ^*OE*^) to generate a novel strain called MBIA (*cat-1* ^*null*^ *::cat-2* ^*OE*^). In this study, we present a thorough characterization of the MBIA (*cat-1* ^*null*^ *::cat-2* ^*OE*^) strain by investigating the body size, reproduction, neuronal health, and dopamine-mediated behaviors.

## Methods

### *C. elegans* husbandry

*C. elegans* were maintained on agar plates with a normal growth medium (NGM) at 20°C with the OP50 strain of *E. coli* used as food. Strains used in this study include N2 (RRID: WB-STRAIN: WBStrain00000001), OK411 (RRID: WB-STRAIN: WBStrain00031419), and UA57 (RRID: WB-STRAIN: WBStrain00035183).

### Synchronization of the population

Synchronization of *C. elegans* populations was performed through the following protocol: Gravid adult *C. elegans* were collected from agar plates using M9 buffer (22 mM KH_2_PO_4_, 42 mM Na_2_HPO_4_, 85 mM NaCl, and 1 mM MgSO_4_ prepared in sterile water) and transferred in 1.5-mL Eppendorf tubes. The tubes were centrifuged at 11,000 rpm for 1 min, the supernatant was removed, and 1 mL of the bleach solution (250 mM KOH and 0.5% hypochlorite prepared in sterile water) was added to each tube. The tubes containing bleach and *C. elegans* were rested on their side with periodic vortexing until most of the bodies disintegrated. The tubes were centrifuged at 11,000 rpm for 1 min, the bleach supernatant was discarded, and the eggs were washed two times to remove any residual bleach solution by resuspending in 1 mL of M9 and centrifuging at 11,000 prm for 1 min. Following the last wash, the supernatant was removed, and the eggs were resuspended in 100 µL of M9 before transferring to 50-mL conical tubes in a final volume of 2 mL M9 solution. Conical tubes were kept on a shaking incubator at 120 rpm at 20°C overnight to let the eggs hatch to the larval 1 stage (L1). L1 *C. elegans* were placed on 6-cm agar growth plates (normal growth medium with live *E. coli* OP50) and placed upside down in a 20°C incubator. For L4 analysis, *C. elegans* samples were collected 48 h after L1 plating. Day 1 adult *C. elegans* were analyzed 72 h after L1 plating. Day 2 adult *C. elegans* were analyzed 96 h after L1 plating. Day 3 adult *C. elegans* were analyzed 120 h after L1 plating.

### Genotype confirmation by PCR

For the confirmation of the genotype of the MBIA strain, polymerase chain reaction (PCR) was performed by collecting ∼5 gravid adult *C. elegans* for analysis using a Sigma Extract-N-AMP™ Tissue PCR Kit (XNAT2) and Apex Taq Red 2× Master Mix. Briefly, *C. elegans* samples were placed in a lysis buffer and incubated in a thermocycler at 55°C for 10 min and 95°C for 3 min. Following incubation, a neutralization solution was added to each tube to halt the reaction. A primer set was used to confirm the gene deletion of *cat-1* with a 701-base pair product in wild-type and a 272-base pair product in *cat-1*-null *C. elegans*. The forward-3 primer used was CCG​CGC​AAA​TGA​ATG​ACC​TA. The reverse-3 primer used was GAA​CCG​GAA​GGT​TCA​AGC​AT. The *C. elegans* lysate was mixed with Apex Taq Red 2× Master Mix, primers, and water and underwent thermocycling at 94°C for 2 min, 94°C (30 s 35×), 55.6°C (30 s 35×), 72°C (1 min 35×), 72°C (5 min), and 4°C (hold). The PCR product was run with a loading buffer on pre-cast 2% gels (Thermo Fisher) using the E-gel Power Snap Electrophoresis System (Thermo Fisher).

### Microscopy analysis

*C. elegans* were imaged on microscope slides with agar pads (3% pure agarose in M9). The agar mixture was brought to a boil before allowing to cool to 58°C. The cooled agar was pipetted onto a glass microscope slide, and a second slide was placed on top to flatten the agar into an even surface. After the agar cooled completely and solidified, the top microscope slide was removed, and 15–30 *C. elegans* were placed on the agar pad in 5 mM levamisole before cover-slipping. The slides were imaged on an EVOS FL or APEX100 microscope using brightfield or GFP at 4×, 10×, and 20× magnification.

#### Body size

*C. elegans* on microscope slides were imaged on an EVOS FL microscope at 4× magnification using brightfield for body size analysis. The images were analyzed using ImageJ and Fiji by using the segmented line tool and drawing a line through the midline of the *C. elegans* from head to tail. The length of the segmented line was recorded in microns as a measurement of the body length of *C. elegans*.

#### Neuron size

*C. elegans* on microscope slides were imaged on an EVOS FL microscope at 20× magnification using GFP at consistent light intensity and exposure. The optimal z-plane was determined by finding the plane where the most neurons in the head were in focus for imaging. ImageJ and Fiji were used to create and apply a macro to consistently analyze neuron area size across different *C. elegans* strains and of different ages by thresholding the images over the fluorescent neurons and recording the neuron area. To exclude any aberrant features during the analysis, the images were manually verified to confirm that the reported areas corresponded to the neurons in the image, and objects with an area value less than 10 square pixels were excluded.

#### Vulval staging

Vulval staging was performed at the L4 stage (48 h post-L1 plating) with precise timing to ensure that all strains were evaluated at the same time post-L1 plating. Vulval stages were observed using brightfield microscopy on the APEX100 microscopy system at 20× magnification and staged based on morphological definitions according to previously established protocols (Mok et al., 2015).

#### Reproduction assay

The reproduction assay was developed to measure the amount of time post-L1 plating until individual *C. elegans* from each strain began laying eggs. Individual L1 *C. elegans* from the synchronized populations were placed one *C. elegans* per well on 24-well plates with agar (normal growth medium) and live OP50. The reproduction assay was performed beginning at the L4 stage (48 h post-L1 plating), and it continued for 24 h. During the 24-h assay, the 24-well plate was imaged in brightfield at 4× magnification in 30-min intervals on the APEX100 microscope system. The images from each well were compiled at the end of the assay and analyzed to determine the time point at which the *C. elegans* began laying eggs. The amount of time post-L1 plating was calculated based on determining the interval between the time the *C. elegans* was plated at L1 and the time the image of the well containing eggs was acquired. Wells were excluded only in the case of loss of *C. elegans* or *C. elegans* viability post-L1 plating or decreased image quality due to loss of the focal plane during acquisition, which prohibited clear analysis.

### Behavior

For behavioral analysis, 4–6 *C. elegans* of a specific genotype and developmental stage were placed in 60 µL of the M9 solution added to microscope slides with hydrophobic circles. A stereomicroscope with a camera was used to analyze the behavior of *C. elegans*. The microscope was focused such that a well-defined image could be captured, and videos of *C. elegans* swimming in the M9 solution were collected for 30,000 ms at 18 fps and saved as JPEG images. For each strain, 4–6 replicates were performed. Behavioral analysis was performed using ImageJ. To perform curling analysis, the image sequence was played through, and the number of times an individual *C. elegans* was in a curled position was counted. We defined curling as the position in which the tail of a *C. elegans* was directly crossing its body (Sohrabi et al., 2021). To perform thrashing analysis, we ensured that animation options were set to 18 fps so that the image sequence played a 30-s video. A stopwatch starting at 0 s was prepared prior to starting the video. The percentage of time for which a *C. elegans* was thrashing over a 30-s time period was measured. We defined thrashing as the motion of a *C. elegans* aggressively moving side to side (Sohrabi et al., 2021).

### Fecundity assay

Individual *C. elegans* were placed in one well of a clear U-shaped 96-well plate filled with 100 µL of a 20% bleach solution (in M9) per well. The plate was observed in brightfield at 4× and 10× using an EVOS FL microscope. Once the *C. elegans* body dissolved and eggs were released, the number of eggs was counted and recorded as a measure of eggs *in utero*.

### COPAS

*C. elegans* at the L4 and day-1 adulthood developmental stages were analyzed using the COPAS FP-250 large particle flow cytometer (Union Biometrica, Massachusetts). *C. elegans* were collected from agar growth plates using 5 mM levamisole to anesthetize the animals. The COPAS cytometer recorded the extinction and time of flight of each object, which was exported in .csv files and analyzed using RStudio. Raw data files from each strain were stored in separate variables and then combined into one .csv file per experiment containing data for all strains using the “rbind” function. A few methods of data cleaning were performed to exclude objects that were not *C. elegans* (e.g., contaminants, pieces of agar, and dust particles). First, the data were analyzed for the number of objects in a sample to determine if excessive contamination was likely due to excessive objects detected and linear data distribution not representative of typical data spread. Any contaminated datasets were removed from analysis. Of the 48 samples compiled for the COPAS analysis, three contaminated samples were removed. Next, the objects were filtered based on extinction measurements to remove objects with extinction values smaller than 250 and greater than 4,000. As reported by other groups, the analysis of particles outside of this range by microscopy revealed objects with extinction values < 250 to be *C. elegans* body fragments, unhatched embryos, pieces of agar, and other contaminants such as dust (Smith et al., 2009). Lastly, any remaining contaminants were removed by determining the average and standard deviation of the compiled strain data. Any values outside the range of mean ±2*StDev were removed. This outlier exclusion resulted in the removal of 4.16% or 423 data points out of 10,158 L4 stage worms and 6.42% or 559 data points out of 8,708 day-1 adulthood stage worms. The EXT and TOF values were imported into GraphPad Prism 10 for statistical analysis, and log-transformations were performed for graphical depiction.

### Statistics

All data were analyzed using the GraphPad Prism 10 statistical software package (San Diego, California). Analysis of more than two groups with discrete variables was done by one-way ANOVA on a mean rank (Kruskal–Wallis [KW]) test with Dunn’s multiple comparisons *post hoc* test. Analysis of more than two groups with continuous variables was done for for normality and lognormality; however, all datasets failed normality tests and, thus, underwent one-way ANOVA on the mean rank (KW) test with Dunn’s multiple comparisons *post hoc* test. Analysis of the neuron area underwent a log-transformation; however, it failed a test for normality and, thus, underwent a Mann–Whitney (MW) *U* test. Data are represented by a truncated violin plot extending to minimum and maximum values, with dashed lines demarking the median and dotted lines demarking quartiles. Groups within each dataset with distinct letter assignments show statistically significant differences between the groups. Groups within each dataset sharing letter assignments show no statistically significant differences between the groups.

## Results

MPTP, through the actions of its toxic metabolite MPP^+^, is a well-known dopaminergic neurotoxicant (Langston, 2017). Animal model data demonstrate specific dopaminergic toxicity resulting from the uptake of MPP^+^ into dopaminergic neurons, where MPP^+^ causes mitochondrial complex I inhibition and displaces dopamine from the synaptic vesicle (Braungart et al., 2004; Meredith and Rademacher, 2011; Langston, 2017; Bradner et al., 2021; Murphy et al., 2021; Mustapha and Mat Taib, 2021). While typically studied for its role in Parkinson’s disease, a disease of aging, MPP^+^ has demonstrated developmental toxicity in *C. elegans.* Treating L1 *C. elegans* with 1.4 mM MPP^+^ for 48 h results in a delay in development, with worms reaching larval stages L3–L4 instead of progressing to adulthood (Braungart et al., 2004). Although we have previously not explored the effect of MPP^+^ treatment on *C. elegans* development, we have observed that the treatment of wild-type L1 *C. elegans* with varying doses of MPP^+^ (0.25 mM–0.75 mM) results in a statistically significant decrease of 16.60% in body size at L4 in worms treated with 0.75 mM MPP^+^ and on day 1 adulthood by 6.40% in worms treated with 0.25 mM MPP^+^ and 16.65% in worms treated with 0.75 mM MPP^+^ (one-way ANOVA on mean ranks with Dunn’s *post hoc* test, n = 48–79 worms per genotype at each developmental stage, *p* < 0.0001), as well as a decrease in eggs *in utero* on day 1 adulthood by an average of 1.10 fewer eggs *in utero* following 0.75 mM MPP^+^ treatment (one-way ANOVA on mean ranks with Dunn’s *post hoc* test, n = 33–39 worms per genotype, *p* < 0.0001) (Supplementary Figure S1). Given these findings, we sought to develop a set of analyses to determine whether body size is a direct correlate of the developmental stage in *C. elegans* or whether the body size and developmental stage are independently regulated. We utilized genetic models of dysregulated dopamine production and storage to develop the following analyses for application in future neurotoxicity experiments.

### Creation of novel *C. elegans* strain “MBIA” overexpressing cat-2 with cat-1 null

**FIGURE 1:**
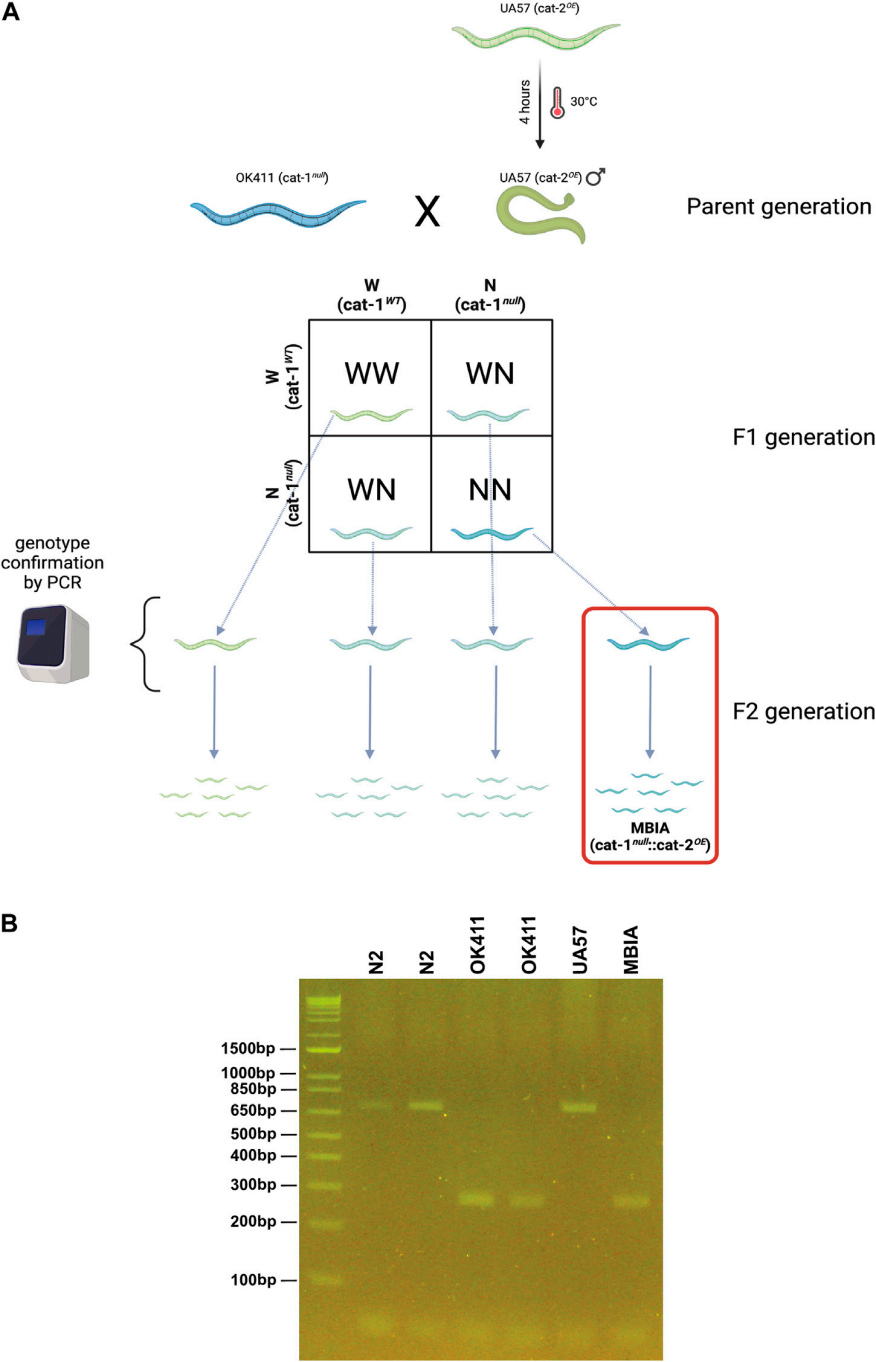
Creation of the novel *C. elegans* strain “MBIA” overexpressing cat-2 with cat-1 null. **(A)** Process of generating the MBIA (cat-1^*null*^::cat-2^*OE*^) strain from parent UA57 (cat-1^*WT*^::cat-2^*OE*^) and OK411 (cat-1^*null*^::cat-2^*WT*^) strains. Generation process consisted of heat-shocking UA57 worms to generate male worms, which were bred with hermaphrodite OK411 worms. Individual F1-generation worms were selected and allowed to reproduce to generate single-genotype lineages. **(B)** PCR from single-genotype lineage populations demonstrating the cat-1 gene product at 701 base pairs for N2 and UA57 strains. Gene deletion resulting in the cat-1-null phenotype results in a 272-base pair gene product in OK411 and MBIA strains. Graphic was created using BioRender.

The novel *C. elegans* strain “MBIA” was generated by crossing the two previously existing strains UA57 (cat-2 overexpressing [*cat-2^OE^*]) and OK411 (cat-1 null [*cat-1^null^*]) (Figure 1A). In addition to overexpressing *cat-2*, an enzyme involved in dopamine synthesis, UA57 worms possess fluorescent dopaminergic neurons expressing the green fluorescent protein (GFP). Male UA57 worms were generated using a heatshock protocol. Briefly, 10–15 L4 hermaphrodite UA57 worms were placed on an agar plate with OP50 food in a 30°C incubator for 4 h. The plate was transferred to a standard incubation environment of 20°C, and the worms were allowed to reproduce. Male UA57 worms were identified by phenotype (e.g., fin-shaped tail) and placed on an agar plate with OP50 food with hermaphrodite worms to generate a consistent source of male UA57 offspring. Male UA57 worms were placed on an agar plate with hermaphrodite OK411 worms to promote breeding to generate the novel MBIA strain containing both the UA57 and OK411 genotypes. Individual first-generation (F1) offspring from the UA57 x OK411 cross with fluorescent dopaminergic neurons indicating the presence of the UA57 genotype were selected and placed on agar plates with OP50 food. These individual F1 worms were allowed to hermaphroditically reproduce, generating second-generation (F2) offspring. Based on the principles of Mendelian inheritance, the F2 offspring were hypothesized to be present at a 1:2:1 ratio of homozygous for wild-type cat-1 alleles, heterozygous for cat-1 alleles, and homozygous for cat-1 null. Thus, individual F2 offspring with fluorescent dopaminergic neurons indicating the presence of the UA57 genotype were selected and placed on agar plates with OP50 food and allowed to reproduce. Once the individual F2 worm had reproduced, the parent F2 worm was collected for PCR analysis to confirm the genotype of the strain. Fluorescent dopaminergic neurons confirmed the presence of the UA57 phenotype, and through PCR analysis, the cat-1 null genotype was identified owing to a shift in genome size (Figure 1B).

### Dysregulation of dopamine synthesis and packaging affects *C. elegans* body length

**FIGURE 2:**
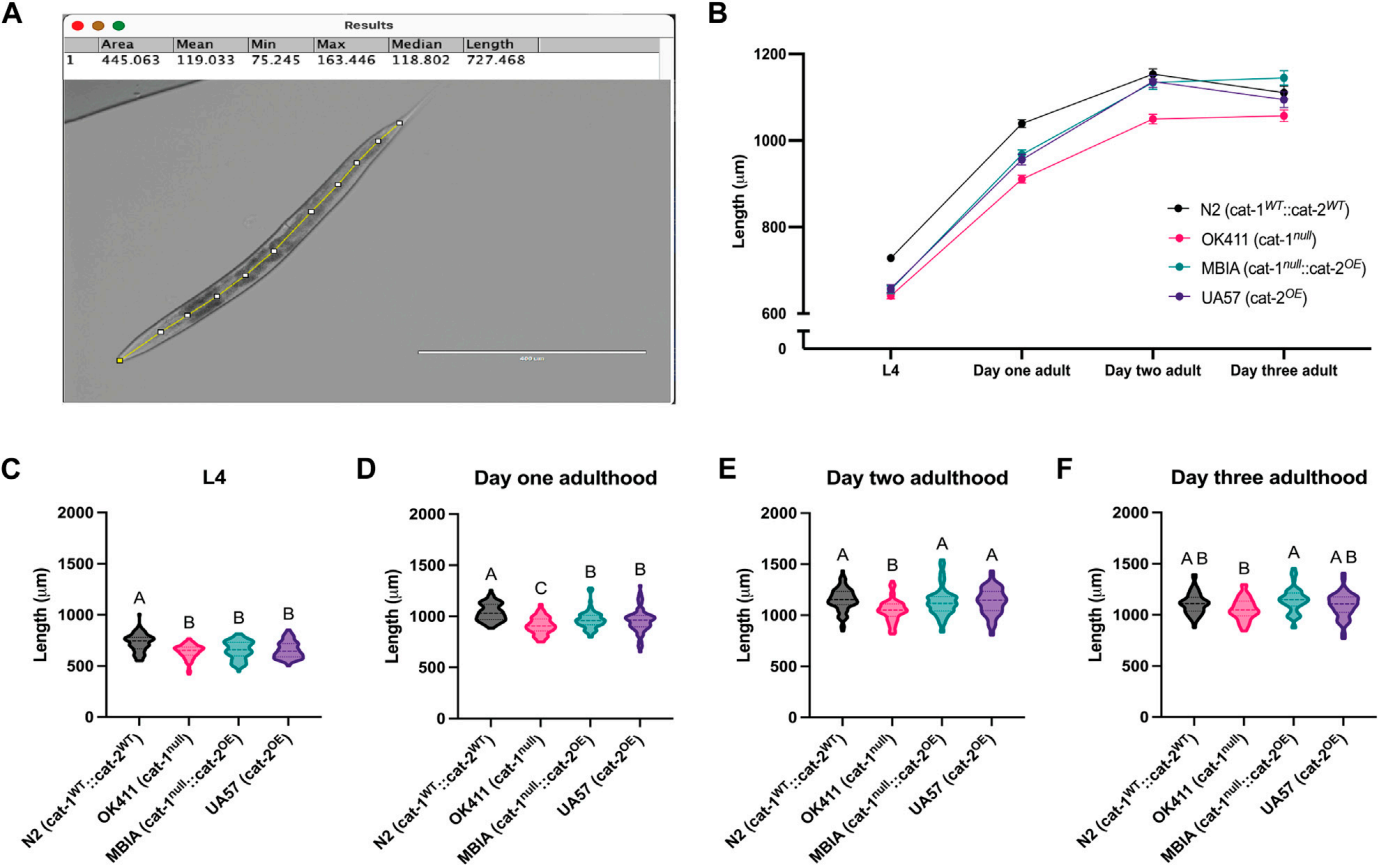
Dysregulation of dopamine synthesis and packaging affects *C. elegans* body length determined by microscopy. **(A)** Representative brightfield image of *C. elegans* used for body length analysis in ImageJ with Fiji with a segmented line through the midline of the animal to generate length measurement. **(B)** Trend of body lengths for wild-type N2 (cat-1^*WT*^::cat-2^*WT*^) in black, OK411 (cat-1^*null*^::cat-2^*WT*^) in pink, MBIA (cat-1^*null*^::cat-2^*OE*^) in teal, and UA57 (cat-1^*WT*^::cat-2^*OE*^) in purple tracked over four developmental stages. **(C–F)** Violin plots with median and quartile demarcations demonstrating the body lengths for each strain at the L4 **(C)**, day-1 adulthood **(D)**, day-2 adulthood **(E)**, and day-3 adulthood **(F)** stages compiled across four experimental replicates, with 47–93 worms per genotype at each developmental stage for each experiment (,244 worms analyzed in total). **(C)** L4. One-way ANOVA on mean ranks, *p* < 0.0001, KW statistics = 50.65. Dunn’s multiple comparisons *post hoc* test: N2 vs. OK411, *p* < 0.0001, Z = 6.444; N2 vs MBIA, *p* < 0.0001, Z = 5.215; N2 vs UA57, *p* < 0.0001, Z = 5.350. **(D)** Day 1 adulthood. One-way ANOVA on mean ranks, *p* < 0.0001, KW statistics = 72.95. Dunn’s multiple comparisons *post hoc* test: N2 vs OK411, *p* < 0.0001, Z = 8.452; N2 vs MBIA, *p* < 0.0001, Z = 4.670; N2 vs UA57, *p* < 0.0001, Z = 5.238; OK411 vs MBIA, *p* < 0.005, Z = 3.429; OK411 vs UA57, *p* < 0.01, Z = 3.189. **(E)** Day 2 adulthood. One-way ANOVA on mean ranks, *p* < 0.0001, KW statistics = 43.24. Dunn’s multiple comparisons *post hoc* test: N2 vs OK411, *p* < 0.0001, Z = 6.111; OK411 vs MBIA, *p* < 0.0005, Z = 4.162; OK411 vs UA57, *p* < 0.0001, Z = 5.002. **(F)** Day 3 adulthood. One-way ANOVA on mean ranks, *p* < 0.0001, KW statistics = 16.77. Dunn’s multiple comparisons *post hoc* test: OK411 vs MBIA, *p* < 0.0005, Z = 4.061.

To determine the effect of dopamine system mutations on body size, *C. elegans* populations were synchronized and allowed to develop to the L4 stage, day-1 adulthood, day-2 adulthood, and day-3 adulthood stages. At each stage, the worms were transferred from the agar culture plate onto microscope slides with agar pads in a solution of levamisole (5 mM) to immobilize the animals for brightfield imaging at 4×. The body length was determined by processing images in ImageJ with Fiji using the segmented line tool. Lines were drawn through the midline of each worm from head to tail, consistently beginning and ending at the same morphological points on each animal and reported in micrometer (Figure 2A). The trend of body lengths for the four strains (Figure 2B) demonstrates body size in the presence of dopamine system mutations over four developmental stages. As the worms continue to develop and age, the overproduction of dopamine begins to rescue the body length deficit partially on day 1 adulthood, with a full rescue in body length to wild-type levels by day 2 adulthood and by day 3 adulthood, nearly equivalent body lengths across all strains. Statistical analysis was performed at each developmental stage to determine whether the differences in body size were statistically significant. N2s showed a statistically significant higher body length over OK411 by 12.01%, MBIA by 10.08%, and UA57 by 9.70% at the L4 stage (Figure 2C) (one-way ANOVA on mean ranks with Dunn’s *post hoc* test, n = 47–93 worms per genotype at each developmental stage, *p* < 0.0001); however, no statistically significant differences were detected in length between the OK411, MBIA, and UA57 strains at the L4 stage. N2 nematodes were significantly longer than OK411 by 12.36%, MBIA by 6.70%, and UA57 by 8.00% at the day-1 adulthood stage (Figure 2D) (one-way ANOVA on mean ranks with Dunn’s *post hoc* test, n = 47–93 worms per genotype at each developmental stage, *p* < 0.0001), and OK411 nematodes were significantly shorter than both MBIA (*p* < 0.01) and UA57 (*p* < 0.01) by 5.87% and 4.73%, respectively, at the day-1 adulthood stage. No statistically significant differences were detected in length between N2, MBIA, and UA57 strains at the day-2 adulthood stage, and OK411 nematodes remained significantly shorter than N2 by 9.02% (Figure 2E) (*p* < 0.0001), MBIA by 7.44% (*p* < 0.0005), and UA57 by 7.64% (*p* < 0.0001). At the day-3 adulthood stage, no statistically significant differences were observed in length among N2, MBIA, and UA57 strains, and OK411 nematodes remained significantly shorter than the MBIA strain by 7.66% (Figure 2F) (*p* < 0.0005).

As a secondary measure of body size, *C. elegans* were analyzed using a COPAS FP-250 large-particle flow cytometer for high-throughput automated measurement. A COPAS-based analysis was included to determine whether a high-throughput method used to measure the body length was able to replicate the findings from the microscopy analysis and to determine whether this method was able to detect more subtle differences in body size due to the larger population analyzed. *C. elegans* were collected at L4 and day-1 adulthood stages for analysis of length. Body size was reported for each worm as time of flight (TOF) indicating length and extinction (EXT) indicating width. Following data processing, detailed in *Methods*, to remove any erroneous objects (e.g., contaminants), the values were compiled across experimental replicates and log-transformed.

**FIGURE 3:**
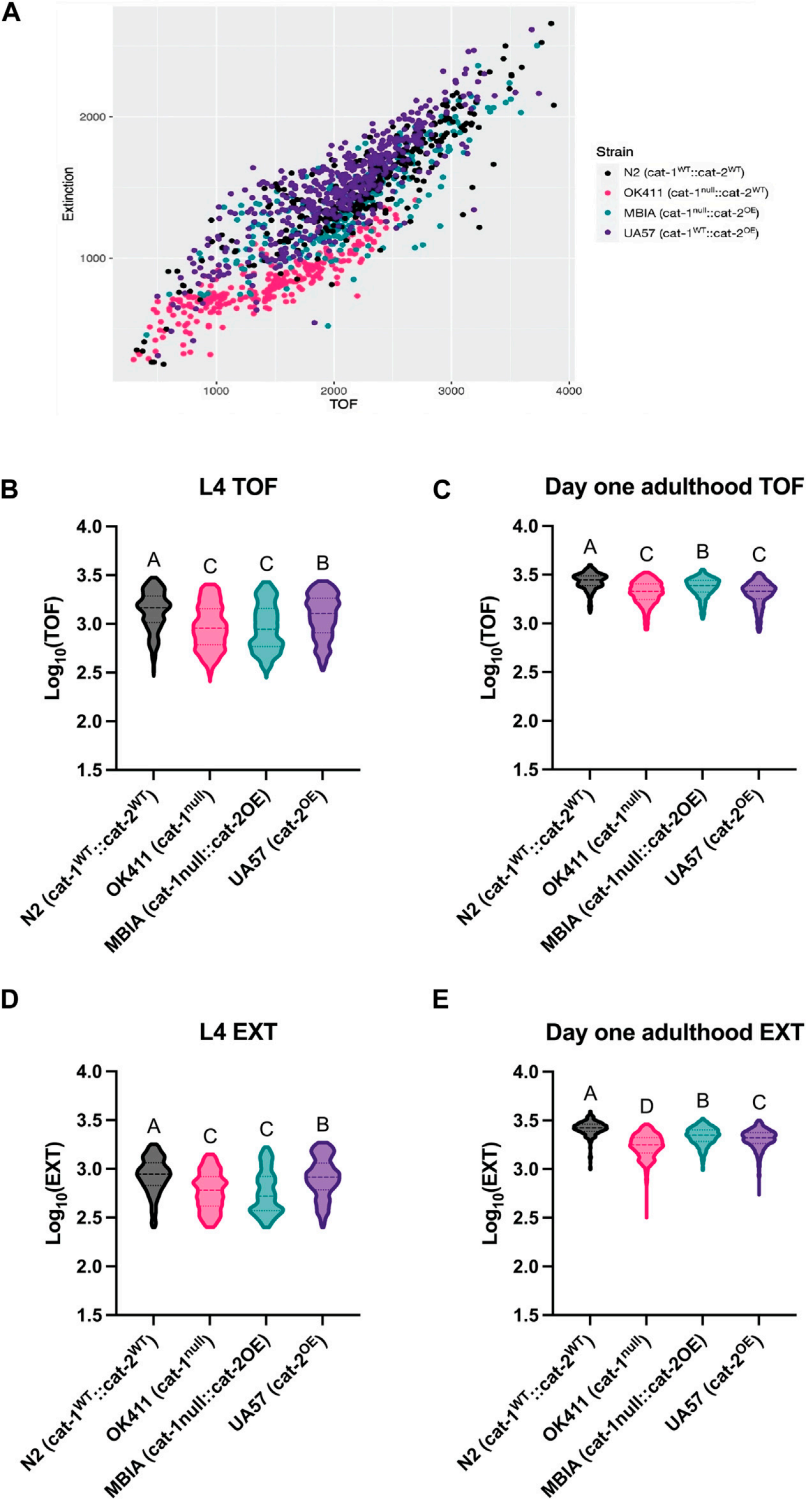
Dysregulation of dopamine synthesis and packaging affects *C. elegans* body length determined by automated analysis. **(A)** Representative TOF vs EXT scatter plot generated from cleaned COPAS data from L4 *C. elegans.* **(B)** Log_10_ (TOF) values for L4 *C. elegans*. One-way ANOVA on mean ranks, *p* < 0.0001, KW statistics = 504.4. Dunn’s multiple comparisons *post hoc* test: N2 vs OK411, *p* < 0.0001, Z = 17.45; N2 vs MBIA, *p* < 0.0001, Z = 18.84; N2 vs UA57, *p* < 0.0001, Z = 6.411; OK411 vs MBIA, ns, Z = 0.2304; OK411 vs UA57, *p* < 0.0001, Z = 11.89; MBIA vs UA57, *p* < 0.0001, Z = 12.96. **(C)** Log_10_ (TOF) values for day-1 adulthood *C. elegans*. One-way ANOVA on mean ranks, *p* < 0.0001, KW statistics = 569.0. Dunn’s multiple comparisons *post hoc* test: N2 vs OK411, *p* < 0.0001, Z = 19.67; N2 vs MBIA, *p* < 0.0001, Z = 10.36; N2 vs UA57, *p* < 0.0001, Z = 20.26; OK411 vs MBIA, *p* < 0.0001, Z = 12.58; OK411 vs UA57, ns, Z = 1.556; MBIA vs UA57, *p* < 0.0001, Z = 13.46. **(D)** Log_10_ (EXT) values for day 1 adulthood *C. elegans*. One-way ANOVA on mean ranks, *p* < 0.0001, KW statistics = 695.2. Dunn’s multiple comparisons *post hoc* test: N2 vs OK411, *p* < 0.0001, Z = 18.30; N2 vs MBIA *p* < 0.0001, Z = 21.10; N2 vs UA57, *p* < 0.001, Z = 3.163; OK411 vs MBIA, *p* < 0.0001, Z = 1.530; OK411 vs UA57, *p* < 0.0001, Z = 15.78; MBIA vs UA57, *p* < 0.0001, Z = 18.52. **(E)** Log_10_ (EXT) values for day-1 adulthood *C. elegans*. One-way ANOVA on mean ranks, *p* < 0.0001, KW statistics = 110.5. Dunn’s multiple comparisons *post hoc* test: N2 vs OK411, *p* < 0.0001, Z = 30.10; N2 vs MBIA, *p* < 0.0001, Z = 13.94; N2 vs UA57, *p* < 0.0001, Z = 18.86; OK411 vs MBIA, *p* < 0.0001, Z = 22.18; OK411 vs UA57, *p* < 0.0001, Z = 15.52; MBIA vs UA47, *p* < 0.0001, Z = 6.599.

The body size of *C. elegans* acquired by the COPAS can be visualized by the scatter plot with an individual worm’s TOF and EXT values, which demonstrates clustering of worms from each genotype (Figure 3A). At the L4 stage, the N2 strain had the largest TOF values compared to OK411 (*p* < 0.0001), MBIA (*p* < 0.0001), and UA57 (*p* < 0.0001) strains (Figure 3B) (one-way ANOVA on mean ranks with Dunn’s *post hoc* test, n = 5,396 total worms compiled across 5–6 experimental replicates, *p* < 0.0001). The OK411 strain had an average TOF value of 68.08% of N2 values, MBIA had an average TOF value of 67.45% of N2 values, and UA57 had an average TOF value of 87.90% of N2 values. While the UA57 strain had statistically significant larger TOF values than OK411 (*p* < 0.0001) and MBIA (*p* < 0.0001), there was no statistically significant difference in TOF values detected between the OK411 and MBIA strains. At the day-1 adulthood stage, the N2 strain again had the largest TOF values compared to OK411 (*p* < 0.0001), MBIA (*p* < 0.0001), and UA57 (*p* < 0.0001) strains (Figure 3C) (one-way ANOVA on mean ranks with Dunn’s *post hoc* test, n = 4,373 total worms compiled across 4–6 experimental replicates, *p* < 0.0001). The OK411 strain had an average TOF value of 76.38% of N2 values, the MBIA strain had an average TOF value of 86.90% of N2 values, and the UA57 strain had an average TOF value of 74.64% of N2 values. However, at the day-1 adulthood stage, the MBIA strain had significantly larger TOF values than OK411 (*p* < 0.0001) and UA57 (*p* < 0.0001), and there were no statistically significant differences in the TOF values detected between OK411 and UA57.

The EXT values at the L4 developmental stage mirrored the TOF values, with the N2 strain having the largest EXT values compared to OK411 (*p* < 0.0001), MBIA (*p* < 0.0001), and UA57 (*p* < 0.001) (Figure 3D) (one-way ANOVA on mean ranks with Dunn’s *post hoc* test, n = 5,390 total worms compiled across 5–6 experimental replicates, *p* < 0.0001). The OK411 strain had an average EXT value of 69.50% of N2 values, the MBIA strain had an average EXT value of 66.99% of N2 values, and the UA57 strain had an average EXT value of 95.28% of N2 values. While the UA57 strain had statistically significantly larger EXT values than OK411 (*p* < 0.0001) and MBIA (*p* < 0.0001), there was no statistically significant difference in EXT values detected between the OK411 and MBIA strains. At the day-1 adulthood stage, the EXT values of each strain were statistically significant from those of all other strains (Figure 3E) (one-way ANOVA on mean ranks with Dunn’s *post hoc* test, n = 4,456 total worms compiled across 4–6 experimental replicates, *p* < 0.0001). The OK411 strain had an average EXT value of 66.07% of N2 values, the MBIA strain had an average EXT value of 83.18% of N2 values, and the UA57 strain had an average EXT value of 77.98% of N2 values. The MBIA strain had statistically significantly larger EXT values than OK411 (*p* < 0.0001) and UA57 (*p* < 0.0001), and the UA57 strain had statistically larger EXT values than OK411 (*p* < 0.0001).

### Dysregulation of dopamine synthesis and packaging affects *C. elegans* vulval development and egg laying

**FIGURE 4:**
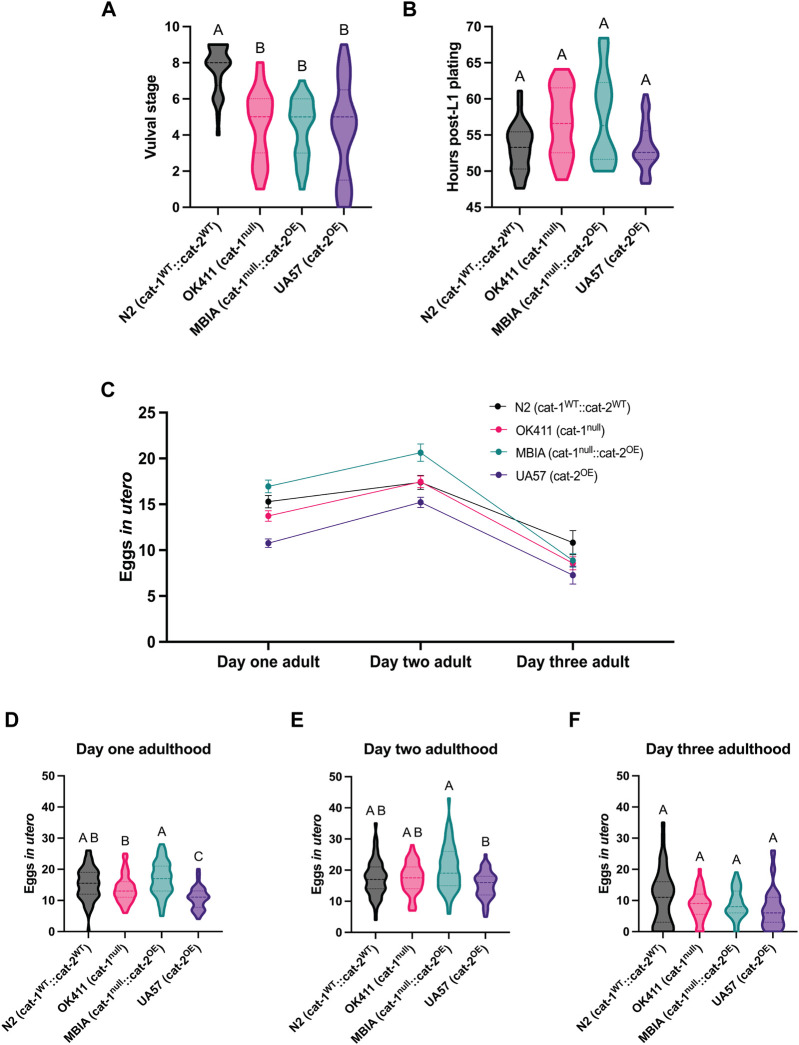
Dysregulation of dopamine synthesis and packaging affects *C. elegans* vulval development and egg laying. **(A)** Vulval stage determined by the morphological analysis of L4 *C. elegans*. One-way ANOVA on mean ranks, *p* < 0.0001, KW statistics = 43.41. Dunn’s multiple comparisons *post hoc* test: N2 vs OK411, *p* < 0.0001, Z = 5.275; N2 vs MBIA, *p* < 0.0001, Z = 5.241; N2 vs UA57, *p* < 0.0001, Z = 5.477. **(B)** Reproduction assay determined by measuring the amount of time in hours post-L1 plating before each genotype began laying eggs. One-way ANOVA on mean ranks, *p* < 0.05, KW statistics = 10.10. Dunn’s multiple comparisons *post hoc* test: n.s. **(C)** Trend of eggs *in utero* for wild-type N2 (cat-1^*WT*^::cat-2^*WT*^) in black, OK411 (cat-1^*null*^::cat-2^*WT*^) in pink, MBIA (cat-1^*null*^::cat-2^*OE*^) in teal, and UA57 (cat-1^*WT*^::cat-2^*OE*^) in purple tracked over three developmental stages. **(D–F)** Violin plot with median and quartile demarcations demonstrating the eggs *in utero* for each genotype at day 1 adulthood **(D)**, day 2 adulthood **(E)**, and day 3 adulthood **(F)** compiled across four experimental replicates with 45–61 *C. elegans* per genotype at each developmental stage for each experiment (650 *C. elegans* analyzed in total). **(D)** Day 1 adulthood. One-way ANOVA on mean ranks, *p* < 0.0001, KW statistics = 47.90. Dunn’s multiple comparisons *post hoc* test: N2 vs UA57, *p* < 0.0001, Z = 5.111; OK411 vs MBIA, *p* < 0.01, Z = 3.276; OK411 vs UA57, *p* < 0.01, Z = 3.286; MBIA vs UA57, *p* < 0.0001, Z = 6.561. **(E)** Day 2 adulthood. One-way ANOVA on mean ranks, *p* < 0.0005, KW statistics = 20.21. Dunn’s multiple comparisons *post hoc* test: MBIA vs UA57, *p* < 0.0001, Z = 4.479. **(F)** Day 3 adulthood. One-way ANOVA on mean ranks, p = n.s., KW statistics = 5.929.

To distinguish whether the observed differences in body size had any effects on development or reproduction, synchronized populations of *C. elegans* were evaluated for vulval development, reproduction timeline, and egg-laying deficits. At the time of analysis (48 h post-L1 plating), the majority of N2 *C. elegans* had fully developed vulvas corresponding to stages 8 and 9; however, the OK411, MBIA, and UA57 strains all showed significantly delayed vulval development at this time point of analysis, with the majority of OK411 at stages 5 and 6, the majority of MBIA at stages 5 and 6, and the majority of UA57 at stages 4 and 5 (Figure 4A) (one-way ANOVA on mean ranks with Dunn’s *post hoc* test, n = 24–32 worms per strain, *p* < 0.0001). To determine whether this delay in vulval development impacted the amount of time post-L1 plating before eggs were laid, a reproduction assay was developed to monitor individual worms for 24 h beginning at L4 to calculate the amount of time post-L1 plating until eggs were laid (Figure 4B). Statistical analysis revealed a significant *p*-value of *p* < 0.05 by one-way ANOVA on mean ranks; however, Dunn’s *post hoc* test did not identify a significant difference in the number of hours until egg laying between any of the strains (one-way ANOVA on mean ranks with Dunn’s *post hoc* test, n = 20–29 worms per strain, *p* < 0.05).

It has been previously reported that *cat-1*-null *C. elegans* have egg-laying deficits, resulting in an increase in eggs *in utero* (Duerr et al., 1999; Bradner et al., 2021). To determine whether overexpression of *cat-2* could rescue egg-laying deficits observed in *cat-1*-null animals, synchronized populations underwent a fecundity assay to quantify the number of eggs *in utero* at day-1 adulthood, day-2 adulthood, and day-3 adulthood stages (Figure 4C). Overall trends show the fewest number of eggs *in utero* in the UA57 strain over multiple days of adulthood and the highest amount of eggs *in utero* in the MBIA strain at day-1 and day-2 adulthood stages. At the day-1 adulthood stage, UA57 had significantly fewer eggs *in utero* than N2 by an average of 4.52 eggs (*p* < 0.0001), OK411 by an average of 2.97 eggs (*p* < 0.01), and MBIA by an average of 6.19 eggs (*p* < 0.0001), and OK411 had significantly fewer eggs *in utero* than MBIA by an average of 3.22 eggs (*p* < 0.01) (Figure 4D) (one-way ANOVA on mean ranks with Dunn’s *post hoc* test, n = 45–61 worms per genotype at each developmental stage). At the day-2 adulthood stage, UA57 had fewer eggs *in utero* than MBIA by an average of 5.41 eggs (*p* < 0.0005), with no other significant differences between strains (Figure 4E) (one-way ANOVA with Dunn’s *post hoc* test, n = 45–61 worms per genotype at each developmental stage). By day-3 adulthood, no statistically significant differences were detected in the number of eggs *in utero* between any strains (Figure 4F) (one-way ANOVA on mean ranks with Dunn’s *post hoc* test, n = 45–61 worms per genotype at each developmental stage, *p* = n.s.).

### Overproduction of dopamine partially rescues deficits in dopamine-mediated behaviors in *C. elegans* with the loss of vesicular dopamine sequestration

**FIGURE 5:**
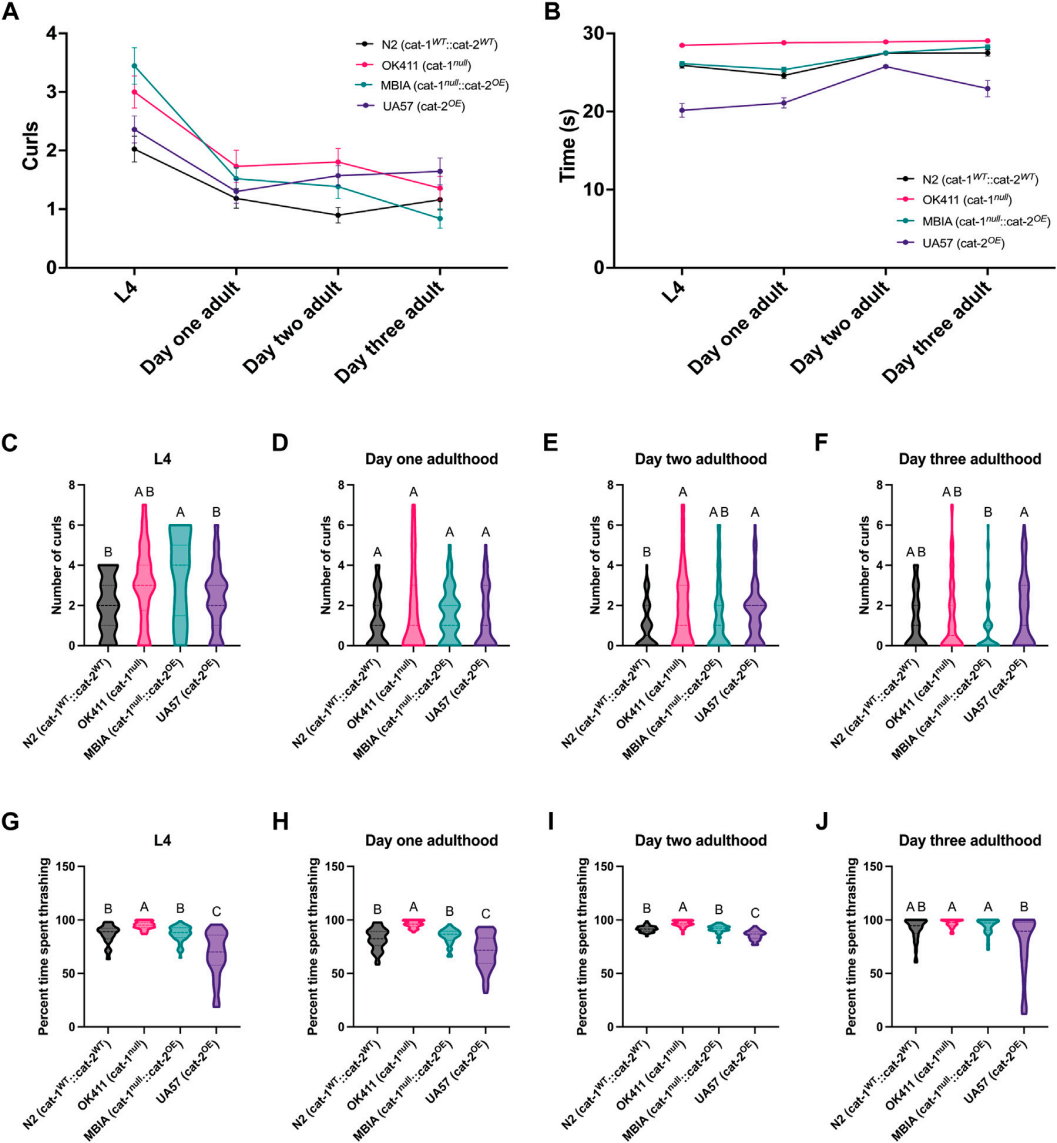
Overproduction of dopamine partially rescues deficits in dopamine-mediated behaviors in *C. elegans* with loss of vesicular dopamine sequestration. **(A)** Trend of curls recorded in 30-s intervals of swimming behavior at four developmental stages with wild-type N2 (cat-1^*WT*^::cat-2^*WT*^) in black, OK411 (cat-1^*null*^::cat-2^*WT*^) in pink, MBIA (cat-1^*null*^::cat-2^*OE*^) in teal, and UA57 (cat-1^*WT*^::cat-2^*OE*^) in purple tracked over four developmental stages. **(B)** Trend of time spent thrashing recorded in 30-s intervals of swimming behavior at four developmental stages. **(C–F)** Violin plot with median and quartile demarcations demonstrating the number of curls for each genotype at L4 **(C)**, day-1 adulthood **(D)**, day-2 adulthood **(E)**, and day-3 adulthood **(F)** stages compiled across three experimental replicates with 955 *C. elegans* analyzed in total. **(C)** L4. One-way ANOVA on mean ranks, *p* < 0.005, KW statistics = 14.63. Dunn’s multiple comparisons *post hoc* test: NS vs MBIA, *p* < 0.005, Z = 3.412; MBIA vs UA57, *p* < 0.05, Z = 2.743. **(D)** Day 1 adulthood. One-way ANOVA on mean ranks, p = n.s., KW statistics = 2.236. **(E)** Day 2 adulthood. One-way ANOVA on mean ranks, *p* < 0.05, KW statistics = 10.99. Dunn’s multiple comparison *post hoc* test: N2 vs OK411, *p* < 0.05, Z = 2.869; N2 vs UA57, *p* < 0.05, Z = 2.732. **(F)** Day 3 adulthood. One-way ANOVA on mean ranks, p = n.s. (0.0593), KW statistics = 7.433. Dunn’s multiple comparisons *post hoc* test: MBIA vs UA57, *p* < 0.05, Z = 2.694. **(G–J)** Violin plot with median and quartile demarcations demonstrating the time spent thrashing for each genotype at L4 **(G)**, day-1 adulthood **(H)**, day-2 adulthood **(I)**, and day-3 adulthood **(J)** stages compiled across three experimental replicates with 884 *C. elegans* analyzed in total. **(G)** L4. One-way ANOVA on mean ranks, *p* < 0.0001, KW statistics = 102.4. Dunn’s multiple comparisons *post hoc* test: N2 vs OK411, *p* < 0.0001, Z = 5.539; N2 vs UA57, *p* < 0.0001, Z = 4.505; OK411 vs MBIA, *p* < 0.0001, Z = 5.674; OK411 vs UA57, *p* < 0.0001, Z = 10.07; MBIA vs UA57, *p* < 0.0001, Z = 4.638. **(H)** Day 1 adulthood. One-way ANOVA on mean ranks, *p* < 0.0001, KW statistics = 120.3. Dunn’s multiple comparisons *post hoc* test: N2 vs OK411, *p* < 0.0001, Z = 7.669; N2 vs UA57, *p* < 0.05, Z = 3.108; OK411 vs MBIA, *p* < 0.0001, Z = 6.677; OK411 vs UA57, *p* < 0.0001, Z = 10.55; MBIA vs UA57, *p* < 0.005, Z = 3.591. **(I)** Day 2 adulthood. One-way ANOVA on mean ranks, *p* < 0.0001, KW statistics = 109.6. Dunn’s multiple comparisons *post hoc* test: N2 vs OK411, *p* < 0.0001, Z = 5.610; N2 vs UA57, *p* < 0.0001, Z = 5.129; OK411 vs MBIA, *p* < 0.0001, Z = 5.061; OK411 vs UA57, *p* < 0.0001, Z = 10.46; MBIA vs UA57, *p* < 0.0001, Z = 5.425. **(J)**. Day 3 adulthood. One-way ANOVA on mean ranks, *p* < 0.0001, KW statistics = 25.57. Dunn’s multiple comparisons *post hoc* test: OK411 vs UA57, *p* < 0.0001, Z = 4.948; MBIA vs UA57, *p* < 0.01, Z = 3.300.

As many behaviors in *C. elegans* have been previously demonstrated as mediated by dopamine, the effect of dopamine-related genetic disruptions on *C. elegans* behavior was measured by quantifying curling and thrashing in 30-s videos of swimming behavior performed at four developmental stages. Curling was defined as the complete overlap of a worm’s tail to a part of its body and quantified as the number of curls in 30-s intervals (Figure 5A) (Sohrabi et al., 2021). Overall trends showed the highest number of curls at L4 in a 30-s interval in the MBIA strain, followed by OK411 and UA57, and the lowest number of curls in N2 (Figure 5A). With development into adulthood, all strains showed fewer curls in a 30-s interval, reaching near equivalent levels in adulthood (Figure 5A). Overall trends showed more time spent thrashing in a 30-s interval in OK411 than in all other strains at L4, with UA57 spending less time thrashing compared to all other strains (Figure 5B). This trend persists generally over time as the worms develop through adulthood (Figure 5B).

Looking at individual differences in each behavior at each developmental stage, at L4, MBIA had, on average, 1.42 more curls than N2 (*p* < 0.01) and 1.08 more curls than UA57 (*p* < 0.05) (Figure 5C) (one-way ANOVA on mean ranks with Dunn’s *post hoc* test, n = 187 total worms compiled across three experimental replicates, *p* < 0.001). At the day-1 adulthood stage, no statistically significant differences were detected between any of the strains in the number of curls (Figure 5D) (one-way ANOVA on mean ranks with Dunn’s *post hoc* test, n = 222 total worms compiled across three experimental replicates, *p* = n.s.). At the day-2 adulthood stage, OK411 had significantly more curling than N2 by an average of 0.91 curls (*p* < 0.05), and UA57 had significantly more curling than N2 by an average of 0.67 curls (*p* < 0.05) (Figure 5E) (one-way ANOVA on mean ranks with Dunn’s *post hoc* test, n = 278 total worms compiled across three experimental replicates, *p* < 0.05). At the day-3 adulthood stage, UA57 had significantly more curling than MBIA by an average of 0.80 curls (*p* < 0.05) despite the one-way ANOVA on mean ranks having a non-significant *p*-value of > 0.05 (Figure 5F) (one-way ANOVA on mean ranks with Dunn’s *post hoc* test, n = 268 total worms compiled across three experimental replicates, *p* = n.s.).

Thrashing was defined as increased spasm-like behavior based on side-to-side movement during swimming behavior and was recorded as the number of seconds spent thrashing during a 30-s interval (Figure 5C) (Sohrabi et al., 2021). At L4, OK411 spent 94.89% of the 30-s interval thrashing, which was significantly higher than that spent by N2 (86.38% of the 30-s interval) (*p* < 0.0001), MBIA (87.05% of the 30-s interval) (*p* < 0.0001), and UA57 (67.16% of the 30-s interval) (*p* < 0.0001) (Figure 5G) (one-way ANOVA on mean ranks with Dunn’s *post hoc* test, n = 220 total worms compiled across three experimental replicates, *p* < 0.0001). No statistically significant differences were detected in the amount of time spent thrashing between N2 and MBIA; however, both N2 and MBIA spent more time thrashing than UA57 (*p* < 0.0001 and *p* < 0.0001, respectively) (Figure 5G) (one-way ANOVA on mean ranks with Dunn’s *post hoc* test, n = 220 total worms compiled across three experimental replicates, *p* < 0.0001). At the day-1 adulthood stage, OK411 continued to display significantly more time spent thrashing, with 96.04% of the 30-s interval spent thrashing compared to N2 (82.07% of the 30-s interval) (*p* < 0.0001), MBIA (84.51% of the 30-s interval) (*p* < 0.0001), and UA57 (70.34% of the 30-s interval) (*p* < 0.0001), and UA57 spent significantly less time thrashing than N2 (*p* < 0.05) and MBIA (*p* < 0.005) (Figure 5H) (one-way ANOVA on mean ranks with Dunn’s *post hoc* test, n = 218 total worms compiled across three experimental replicates, *p* < 0.0001). This trend continued at the day-2 adulthood stage, where OK411 displayed significantly more time thrashing, with 96.39% of the 30-s interval spent thrashing compared to N2 (91.60% of the 30-s interval) (*p* < 0.0001), MBIA (91.69% of the 30-s interval) (*p* < 0.0001), and UA57 (85.85% of the 30-s interval) (*p* < 0.0001), and UA57 spent significantly less time thrashing than N2 (*p* < 0.001) and MBIA (*p* < 0.0001) (Figure 5I) (one-way ANOVA on mean ranks with Dunn’s *post hoc* test, n = 200 total worms compiled across three experimental replicates, *p* < 0.0001). At the day-3 adulthood stage, the only significant differences between the genotypes were significantly more time spent thrashing in OK411, with 96.81% of the 30-s interval spent thrashing, compared to the UA57 (76.44% of the 30-s interval) (*p* < 0.0001), and less time spent thrashing in the UA57 compared to MBIA (94.17% of the 30-s interval) (*p* < 0.01) (Figure 5J) (one-way ANOVA on mean ranks with Dunn’s *post hoc* test, n = 246 total worms compiled across three experimental replicates, *p* < 0.0001).

### Overproduction of dopamine does not affect neuron size in *C. elegans* with the loss of vesicular dopamine sequestration in early adulthood

**FIGURE 6:**
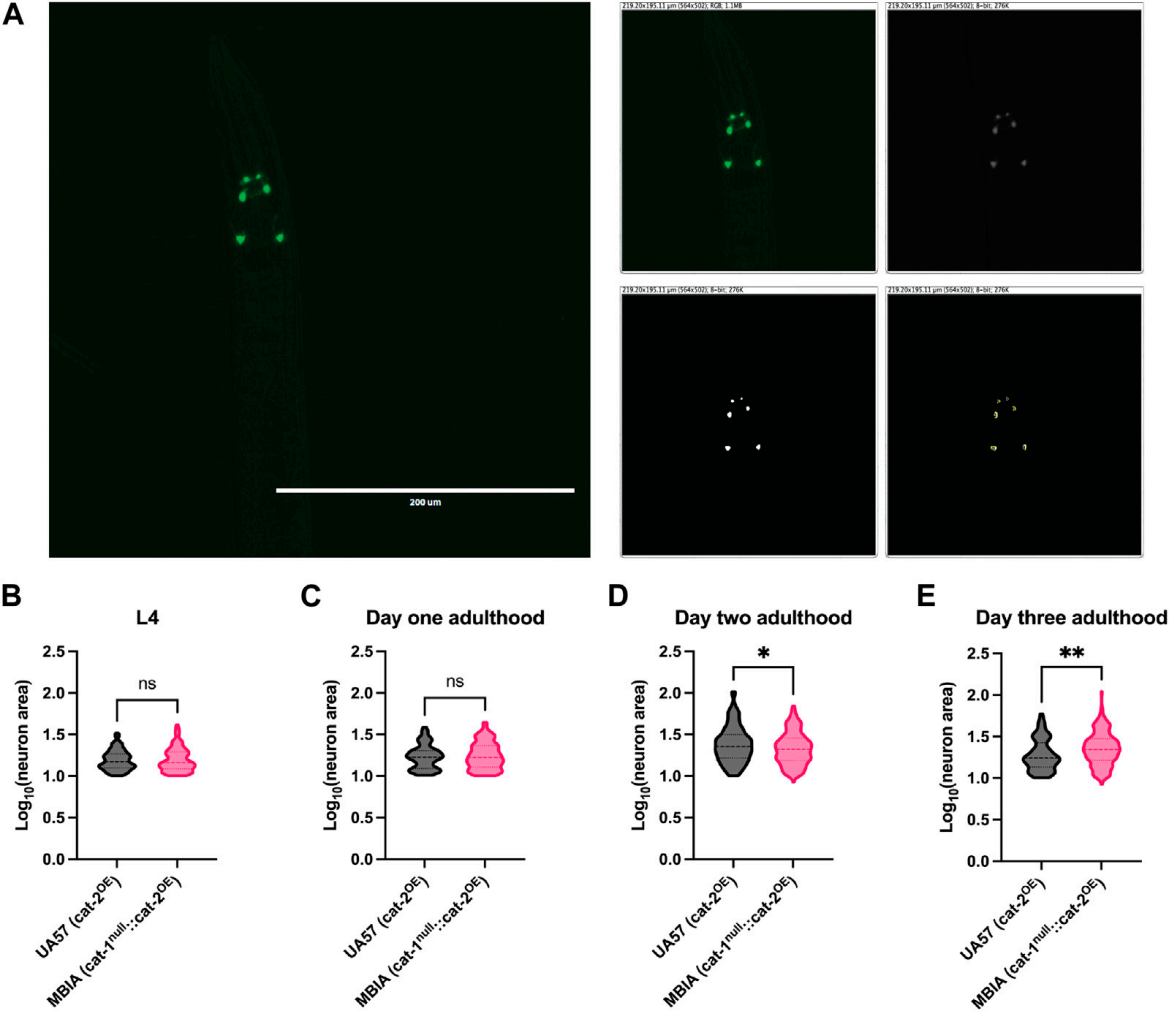
Overproduction of dopamine does not affect neuron size in *C. elegans* with the loss of vesicular dopamine sequestration in early life. **(A)** Representative 20× GFP image of dopaminergic neurons in the head of *C. elegans* used for neuron size analysis in ImageJ with Fiji. Image processing of the original image (top left) demonstrating conversion to an 8-bit image (top right), thresholding (bottom left), and ROI size analysis (bottom right). **(B–E)** Violin plot with median and quartile demarcations demonstrating the neuron size for each genotype at L4 **(B)**, day-1 adulthood **(C)**, day-2 adulthood **(D)**, and day-3 adulthood **(E)** stages compiled with 1,038 neurons in total measured across four experimental replicates. **(B)** L4. MW test, *p* = n.s., U = 8,853. **(C)** Day 1 adulthood. MW test, *p* = n.s., U = 5,331. **(D)** Day 2 adulthood. MW test, *p* < 0.05, U = 19,714. **(E)** Day 3 adulthood. MW test, *p* = <0.005, U = 7,729.

Behavioral deficits resulting from dopamine dysfunction may be due to deficient dopaminergic neurotransmission or the degeneration of dopaminergic neurons. To determine whether the observed behavioral deficits resulted from differences in neuronal health, the size of the dopaminergic neurons was quantified using GFP images of UA57 and MBIA *C. elegans* from the L4 to day-3 adulthood stages. Neuron size was determined by processing GFP images in ImageJ with Fiji using the thresholding and ROI area tools. Thresholding was performed to create a mask over dopaminergic neurons, and the area of the ROI was quantified (Figure 6A). A log-transformation was performed on the raw neuron area values to improve data visualization. No statistically significant differences were observed in neuronal size observed at L4 (MW test, *p* = n.s., U = 8,853) (Figure 6B) and day-1 adulthood stages (MW test, p = n.s., U = 5,331) (Figure 6C); however, at the day-2 adulthood stage, the MBIA neurons were significantly smaller than UA57 by 3.56% (Figure 6D) (MW test, *p* < 0.05, U = 19,714), and at the day-3 adulthood, the MBIA neurons were significantly larger than UA57 neurons by 5.62% (MW test, *p* < 0.005, U = 7,729) (Figure 6E).

## Discussion

Evidence from genetic and toxicologic studies has implicated dopaminergic signaling in the regulation of body size, development, and behavior in *C. elegans*. Prior studies discovered that *C. elegans* with the *cat-2^null^* genotype, lacking the ability to catalyze the production of L-DOPA, had increased body size compared to wild-type animals (Nagashima et al., 2016). Interestingly, despite this study demonstrating an increase in body size due to the lack of dopamine synthesis, we have anecdotally observed smaller body sizes in *C. elegans* with the *cat-1* ^null^ phenotype that have normal dopamine production but lack the protein responsible for vesicular sequestration of dopamine. Furthermore, we have observed a smaller body size in wild-type *C. elegans* exposed to the dopaminergic neurotoxicant MPP^+^ (Supplementary Figure S1). In addition to our observations of MPP^+^-induced decreases in body size, other groups have observed that impairments in development through larval stages into gravid adulthood follow MPP^+^ exposure (Braungart et al., 2004). Building on our previous work that compared toxicant-exposed *C. elegans* to genetic models of dysregulated dopamine systems, we sought to better characterize and understand the effect of altered dopamine production and storage on the development of *C. elegans* (Bradner et al., 2021).

We first performed a thorough analysis of body size by quantifying body length through two independent measures—microscopy and automated high-throughput COPAS analysis. Microscopy analysis was performed on C. elegans up to the day-3 adulthood stage by transferring synchronized adult worms to new agar plates during each day of adulthood after egg laying began to ensure that no future generations of worms were included in the analysis. Based on the method of collecting *C. elegans* and the number of animals analyzed using the COPAS, analysis was only performed at L4 and day-1 adulthood stages to prevent the inclusion of future generations of worms in the analysis.

The analysis of body size using microscopy shown in Figure 2 demonstrated an overall trend that OK411 *C. elegans* lacking the *cat-1* protein have significantly shorter body lengths than wild-type N2 *C. elegans* throughout development and persisting into fully developed adulthood stages (Figures 2, 3). In comparison, although the UA57 and MBIA strains initially display smaller body size at L4, by the day-1 adulthood stage, there was a partial rescue in body size, which reaches wild-type N2 levels by the Day-2 adulthood stage (Figure 2). Furthermore, no statistically significant differences were detected in body size between the MBIA and UA57 strains at any developmental stage, suggesting that the endogenous overproduction of dopamine rescues the *cat-1^null^* phenotype, resulting in improvements in body length, as determined by microscopy.

In comparison to the microscopy data, the COPAS data provide an insight into subtle differences between strains, likely due to the increased population size able to be analyzed by high-throughput measures in comparison to the manual microscopy analysis. Additionally, the COPAS analysis provides both measures of body length (TOF) and width (EXT). At L4, our data show significant differences in body size by TOF and EXT, with the N2 strain having the largest average values, followed by UA57, and finally by OK411 and MBIA (Figures 3B, D). This distribution of data suggests a deficit in TOF and EXT, corresponding to body length and width, respectively, due to both the *cat-2^OE^* and *cat-1^null^* mutations, with a larger effect due to the *cat-1^null^* mutation present in the OK411 and MBIA strains. However, by the day-1 adulthood stage, the TOF and EXT values for the MBIA strain are closer to N2 levels than OK411 and UA57, suggesting that the combination of *cat-1^null^* and *cat-2^OE^* variants can partially rescue the deficits in body size closer to wild-type N2 levels (Figures 3C, E). The overall trends observed in both the microscopy and COPAS analysis data suggest a persistent decrease in body size due to the *cat-1^null^* genotype in the OK411 strain, which is rescued in the MBIA strain to near wild-type N2 levels in the COPAS data and wild-type N2 levels in the microscopy data through introducing the *cat-2^OE^* genotype.

To determine whether the observed differences in body length were a result of developmental delay, we investigated multiple measures of *C. elegans* development and reproduction: vulval staging, development to gravid adulthood, and egg laying. Vulval staging was performed by morphologically determining the stage of development of the vulva and defining the stage across a spectrum from L4.0 to L4.9, corresponding to sequential stages of development. Other groups who have investigated the development of the vulva in *C. elegans* have determined the amount of time it takes to develop through each vulval stage to reach the final stages of development, corresponding to stages L4.8 and L4.9 (Mok et al., 2015). Analysis performed at 48 h post-L1 plating demonstrated more instances of a fully developed vulva in N2 compared to fewer fully developed vulva in OK411, MBIA, and UA57 (Figure 4A), suggesting a slight delay in the development of the vulva due to mutations in dopamine-related proteins. Our data suggest that the OK411, MBIA, and UA57 strains all progressed through the developmental stages at a slower rate than the N2 strain.

In addition to the observed delay in development to fully formed vulva in the dopamine mutants, the number of hours post-L1 plating before egg laying was observed and tested in all strains. Despite the difference in vulval development observed at 48 h post-L1 plating, no statistically significant differences were observed in the number of hours post-L1 plating when egg laying was observed between any of the strains (Figure 4B). Taken together, these data suggest that although the development through the vulval stages may be delayed in the OK411, MBIA, and UA57 strains, this does not impact the amount of time until the strains lay eggs.

The body size, development, and reproduction data have significant implications for the design of experiments, which depend on *C. elegans* body size and developmental stage. Body size differences on the magnitude of tens to hundreds of microns as reported in our dopamine mutant strains can result in misassignment of the developmental stage. The body size data given in Figures 2, 3 in combination with the developmental data given in Figure 4 demonstrate that despite a smaller body size measured in the OK411 strain, these *C. elegans* reach gravid adulthood at the same time as wild-type N2 *C. elegans.* Thus, body size cannot always be used as a proxy for the developmental stage. The COPAS biosorter typically uses the distribution of TOF and EXT to define and sort *C. elegans* based on developmental stages (Boyd et al., 2012). Given that our data demonstrate that OK411 *C. elegans* show a persistent smaller body size throughout all developmental stages analyzed, it is important for experimenters to ensure there are no body size deficits resulting from genetic variation or pharmacological or toxicological treatment in experiments that utilize the COPAS biosorter for analysis or sorting of animals.

Although previous studies have reported potential egg-laying deficits in *cat-1-null* *C. elegans,* as evidenced by an increase in the number of eggs *in utero*, this was not observed in our analysis (Figures 4C–F). Our analysis at the day-1 adulthood stage showed an equivalent number of eggs *in utero* between N2 and MBIA strains, with fewer eggs *in utero* in OK411 and UA57 strains (Figure 4D). However, although statistically significant, the difference in the number of eggs *in utero* is relatively small, with an average of 3.22 fewer eggs in OK411 than in MBIA, an average of 4.52 fewer eggs in UA57 than in N2, and an average of 6.19 fewer eggs in UA57 than in MBIA. A difference of a few eggs *in utero* (≤3) may not be biologically relevant and within a normal range of variation; however, for differences of larger magnitudes, for example, the difference of an average of 6.19 eggs *in utero* between UA57 and MBIA, it is likely that this is biologically relevant and could result from a delay in development to gravid adulthood, resulting in fewer eggs being produced, as evidenced by the slight delay in vulval development shown in Figure 4A. Despite a possible delay in development to gravid adulthood, the reproduction assay demonstrated that all worm strains investigated lay eggs by the day-1 adulthood stage. By the day-2 adulthood stage, the UA57 strain had fewer eggs *in utero* compared to the MBIA strain by an average of 5.41 eggs *in utero*, while no statistically significant differences were detected between N2, OK411, and MBIA strains (Figure 4E). As egg laying has been shown to be mediated by dopamine, it is possible that the UA57 strain has enhanced egg-laying behavior, resulting in fewer eggs *in utero*, explaining the data from the day-1 and day-2 adulthood stages (Figures 4D, E) (Weinshenker et al., 1995). By the day-3 adulthood stage, there is an overall decrease in the number of eggs *in utero* across all strains, resulting in no statistical differences being detected.

To further understand the impact of gene variants on dopamine-related proteins, two additional behaviors (e.g., curling and thrashing during swimming) were analyzed as measures of dopamine-mediated behavior. Overall trends demonstrate an increase in curling behavior in the strains with the *cat-1^null^* phenotype (OK411 and MBIA) at L4, with a decrease in the number of curls in a 30-s interval toward wild-type N2 levels through development (Figures 5A, C–F). Although the differences in the average number of curls per 30-s interval are statistically significant, the differences are of small magnitude, with the average difference in the number of curls ranging from <1 to 1.5 curls. Although this may be biologically relevant, future studies with more sensitive measures of behavior may be able to detect larger differences in behaviors between the strains.

In comparison to the curling data, the thrashing data show an overall trend toward an inverse relationship between the OK411 and UA57 strains, where the OK411 strain displays the highest percent of time spent thrashing during a 30-s interval and the UA57 strain shows the least amount of time spent thrashing during a 30-s interval (Figure 5B). In comparison, the MBIA strain performs equivalently to wild-type N2 levels in terms of the amount of time spent thrashing across all four developmental stages tested, suggesting a rescue to wild-type levels of thrashing from the combination of dopamine system mutations in the MBIA strain (Figures 5B, G–J). The values measured for time spent thrashing in a 30-s interval showed a wider range of values in the UA57 strain compared to the other strains, with an increased number of *C. elegans* that spent less time thrashing. This may be a result of the phenomenon of swimming-induced paralysis (SWIP), which is a well-demonstrated behavior in which *C. elegans* with excess synaptic dopamine levels show inhibited locomotion, particularly with swimming behaviors (Kudumala, Sossi et al., 2019). Taken together, the behavioral data from OK411 and UA57 provide evidence of dopamine-mediated thrashing behavior, where less dopamine transmission resulting from *cat-1* null results in increased thrashing, whereas increased dopamine transmission resulting from *cat-2* overexpression causes less thrashing. This suggests that the endogenous overproduction of dopamine resulting from *cat-2* overexpression is able to rescue the effects of *cat-1* null in *C. elegans*.

It is known that dysregulated dopamine can be neurotoxic and lead to the degeneration of dopaminergic neurons in rodent and *C. elegans* models (Caudle et al., 2007; Bucher et al., 2020; Bradner et al., 2021). While most of these models require aging of animals to observe neurodegeneration, neuronal health analysis was performed to determine whether the observed behavioral effects were a result of neurodegeneration. Analysis was performed only on the UA57 and MBIA animals as these animals express GFP in their dopaminergic neurons, allowing for the quantification of neuron size, and the N2 and OK411 animals do not express GFP in their dopaminergic neurons. While there were no observed differences in the neuronal area between the two strains at L4 and day-1 adulthood stages, the day-2 adulthood stage showed a slight decrease in the log-transformed values for neuron size in the MBIA strain compared to the UA57 strain (Figure 6D) and a slight decrease in the log-transformed neuron size in the UA57 strain compared to the MBIA strain at the day-3 adulthood stage (Figure 6E). Despite these differences existing in the log-transformed data, analysis of the raw values of neuronal area revealed only a significant difference in neuron size at the day-3 adulthood stage, with UA57 showing a smaller neuronal area of 14.68% compared to MBIA (data not shown).

Both the overproduction of dopamine and the lack of dopamine sequestration independently result in increased cytosolic dopamine; thus, it is possible that the MBIA animals would have exacerbated neuronal vulnerability due to the compounded increase in cytosolic dopamine resulting from both *cat-1* null and *cat-2* overexpression. Furthermore, as dysregulated dopamine and interactions between dopamine and alpha-synuclein are involved in the pathogenic degeneration of dopaminergic neurons in Parkinson’s disease, additional experiments are warranted to thoroughly characterize both pathological markers of neuronal health and neurodegeneration resulting in the MBIA strain. For instance, other groups have reported increased dopaminergic neurotoxicity in *cat-2* overexpression in *C. elegans* that express the pathogenic A53T alpha-synuclein mutation; however, this toxicity is ameliorated by mutating the amino acid residues on alpha-synuclein, where it is hypothesized that dopamine binds (Mor et al., 2017). Thus, future experiments will be dedicated to investigating these markers of neuronal health and investigating the consequence of aging on neuronal health in this strain.

Taken together, we sought to determine whether increasing the synthesis of dopamine through *cat-2* overexpression could rescue any deficits observed in the *cat-1*-null genotype. Thus, we created a novel strain (MBIA) with both the *cat-1*-null and *cat-2* overexpression genotypes. The characterization of the MBIA strain has demonstrated that abnormalities resulting from deficient vesicular sequestration of dopamine can be rescued, as shown by the body length and behavioral similarities between the MBIA strain and wild-type. However, the endogenous overproduction of dopamine in the MBIA strain could not rescue delays in development resulting from the loss of dopamine sequestration in the OK411 strain, as observed by vulval staging. Despite the delay in the development to gravid adulthood, the MBIA strain appears to reproduce normally based on egg *in utero* analysis. The ability of the endogenous overproduction of dopamine to rescue the sequestration deficits can possibly be explained by a mechanism by which the excess production of dopamine in the cytosol of MBIA dopaminergic neurons can exit the neuron through non-evoked release mechanisms, thereby achieving synaptic transmission and action at post-synaptic receptors. For instance, a previous work has shown a reversal of the plasmalemmal dopamine transporter (DAT), which typically facilitates the presynaptic reuptake of dopamine from the synapse, but in the cases of excess cytosolic dopamine, such as resulting from amphetamine treatment, can efflux dopamine from the cytosol into the synapse (Kahlig et al., 2005).

Our goal was to develop a set of analyses to measure *C. elegans* body size, developmental stage, and reproduction to determine whether decreases in body size as a result of toxicant exposure and/or genetic manipulation were solely an effect on the size of the animal or an effect on the development through larval stages into gravid adulthood and the ability to reproduce. The characterization of dopamine-related mutations of body size, development, and behavior presented here can be used as a baseline to which *C. elegans* exposed to pharmacological and toxicological compounds can be compared to identify compounds that modulate dopamine signaling.

## Data Availability

The datasets presented in this study can be found in online repositories. The names of the repository/repositories and accession number(s) can be found at: https://doi.org/10.5061/dryad.hhmgqnkpf.

## Supplementary material

SUPPLEMENTARY FIGURE S1: Developmental exposure to MPP^+^ results in decreased body size and fewer eggs *in utero*. **(A)** Wild-type N2 *C. elegans* were plated at L1 on agar plates treated with DMSO (1.5%) and 0.25 mM, 0.5 mM, or 0.75 mM MPP^+^ for analysis at L4 (48 h post-L1 plating) and day-1 adulthood (72 h post-L1 plating) stages. The trend of body lengths for DMSO (1.5%) is shown in green, 0.25 mM MPP^+^ in dark purple, 0.5 mM MPP^+^ in light purple, and 0.75 mM MPP^+^ in blue. **(B,C)** Violin plot with median and quartile demarcations demonstrating the body lengths for each treatment at the L4 **(B)** and day-1 adulthood **(C)** compiled across four experimental replicates with 48–79 worms per treatment at each developmental stage. **(B)** L4. One-way ANOVA of mean ranks, *p* <0.0001, Kruskal–Wallis statistics = 30.72. Dunn’s multiple comparisons *post hoc* test: DMSO (1.5%) vs 0.75 mM MPP^+^, *p* <0.0001, Z = 5.195. **(C)** Day 1 adulthood. One-way ANOVA of mean ranks, *p* <0.0001, Kruskal–Wallis statistics = 49.48. Dunn’s multiple comparisons *post hoc* test: DMSO (1.5%) vs 0.25 mM MPP^+^, *p* <0.001, Z = 2.994; DMSO (1.5%) vs 0.75 mM MPP^+^, *p* <0.0001, Z = 6.123. **(D)** Violin plot with median and quartile demarcations demonstrating the eggs *in utero* for each treatment at the day-1 adulthood stage compiled across four experimental replicates with 33–39 *C. elegans* per treatment. One-way ANOVA on mean ranks, *p* <0.0001, Kruskal–Wallis statistics = 24.87. Dunn’s multiple comparisons *post hoc* test: DMSO (1.5%) vs 0.75 mM MPP^+^, *p* <0.05, Z = 2.570.
