# Supplementary figures and images for: Effect of altered production and storage of dopamine on development and behavior in C. elegans
Source: Front Toxicol. 2024 Aug 16;6:1374866. doi: 10.3389/ftox.2024.1374866 (PMC11363549; doi:10.3389/ftox.2024.1374866)

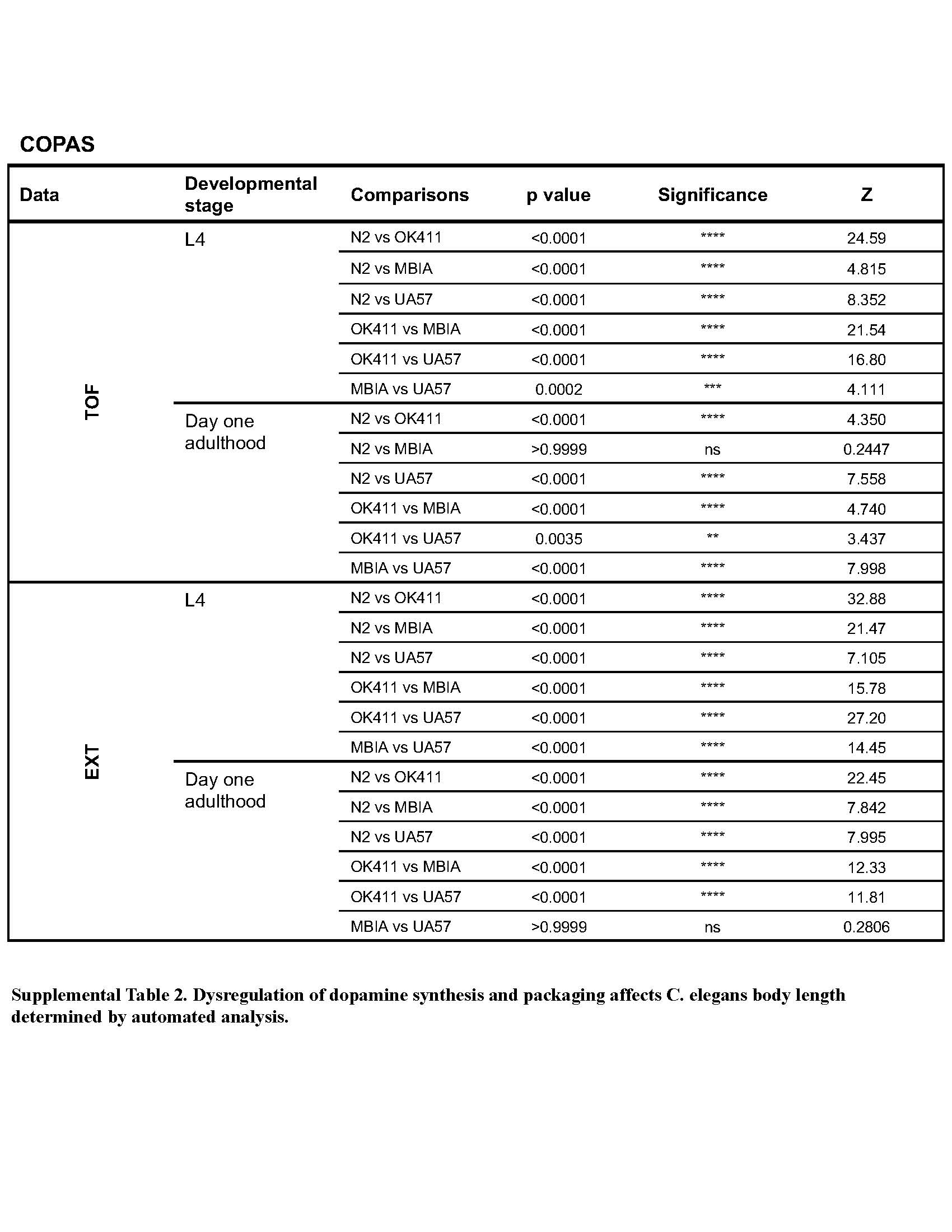

Supplement: Supplementary file 1 [file Image3.JPEG]

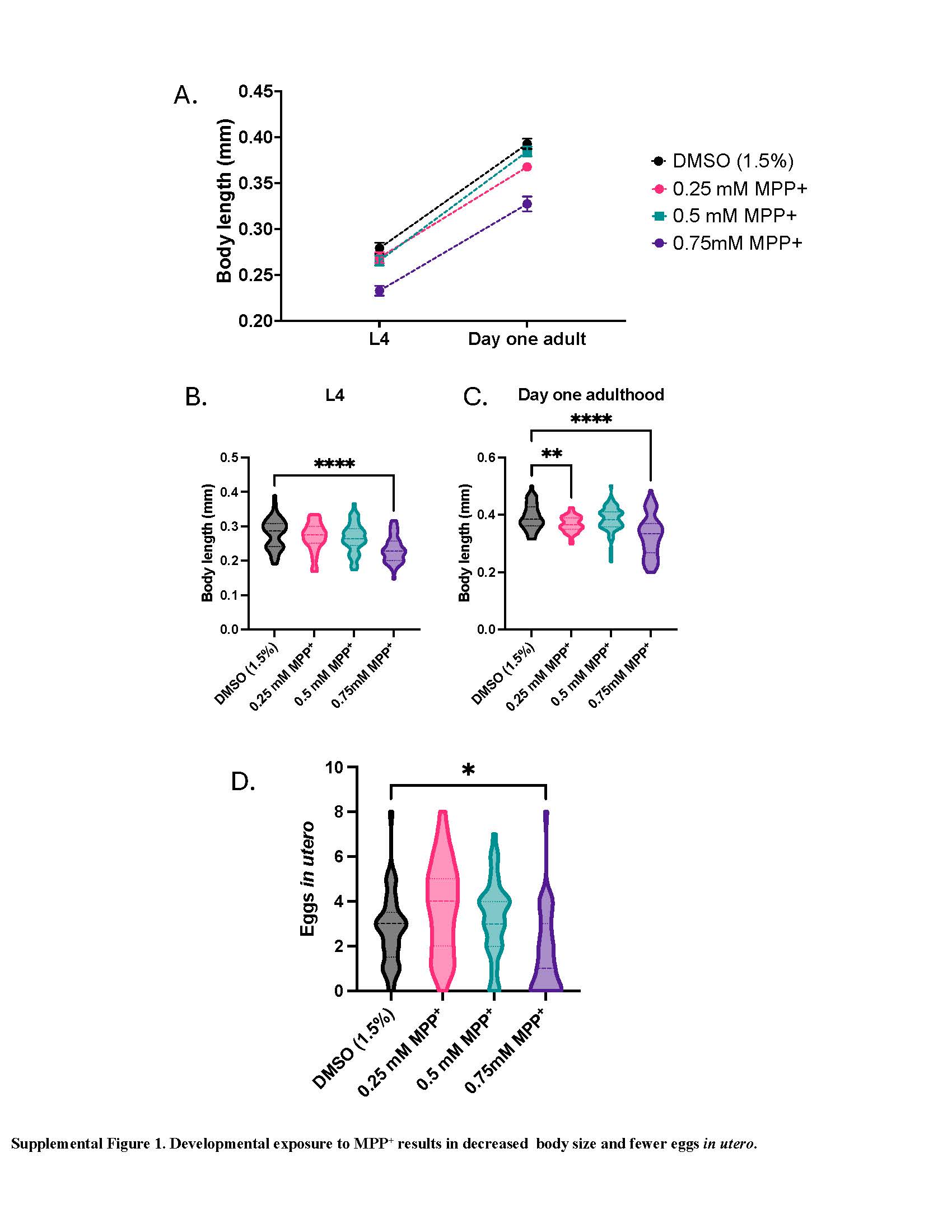

Supplement: Supplementary file 2 [file Image1.JPEG]

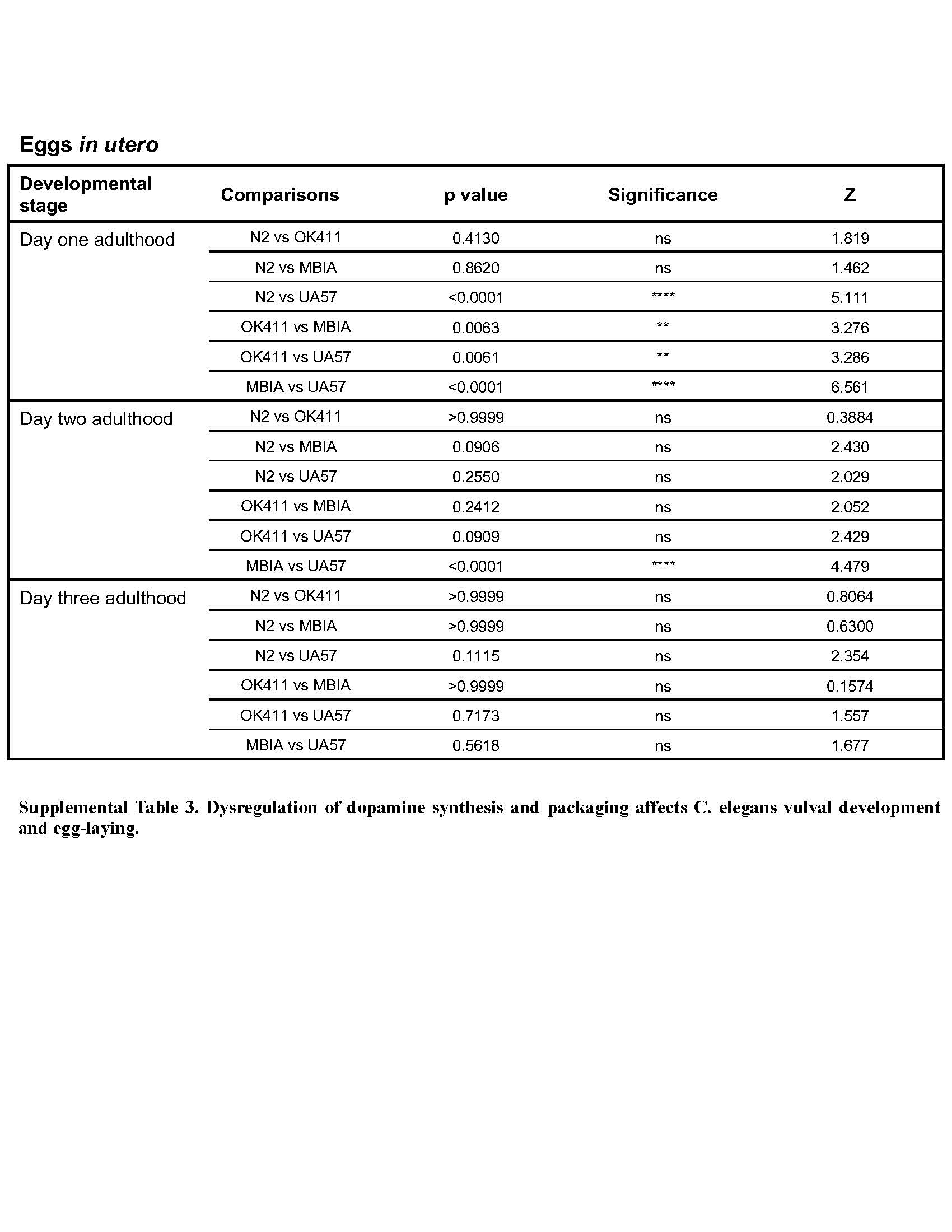

Supplement: Supplementary file 3 [file Image4.JPEG]

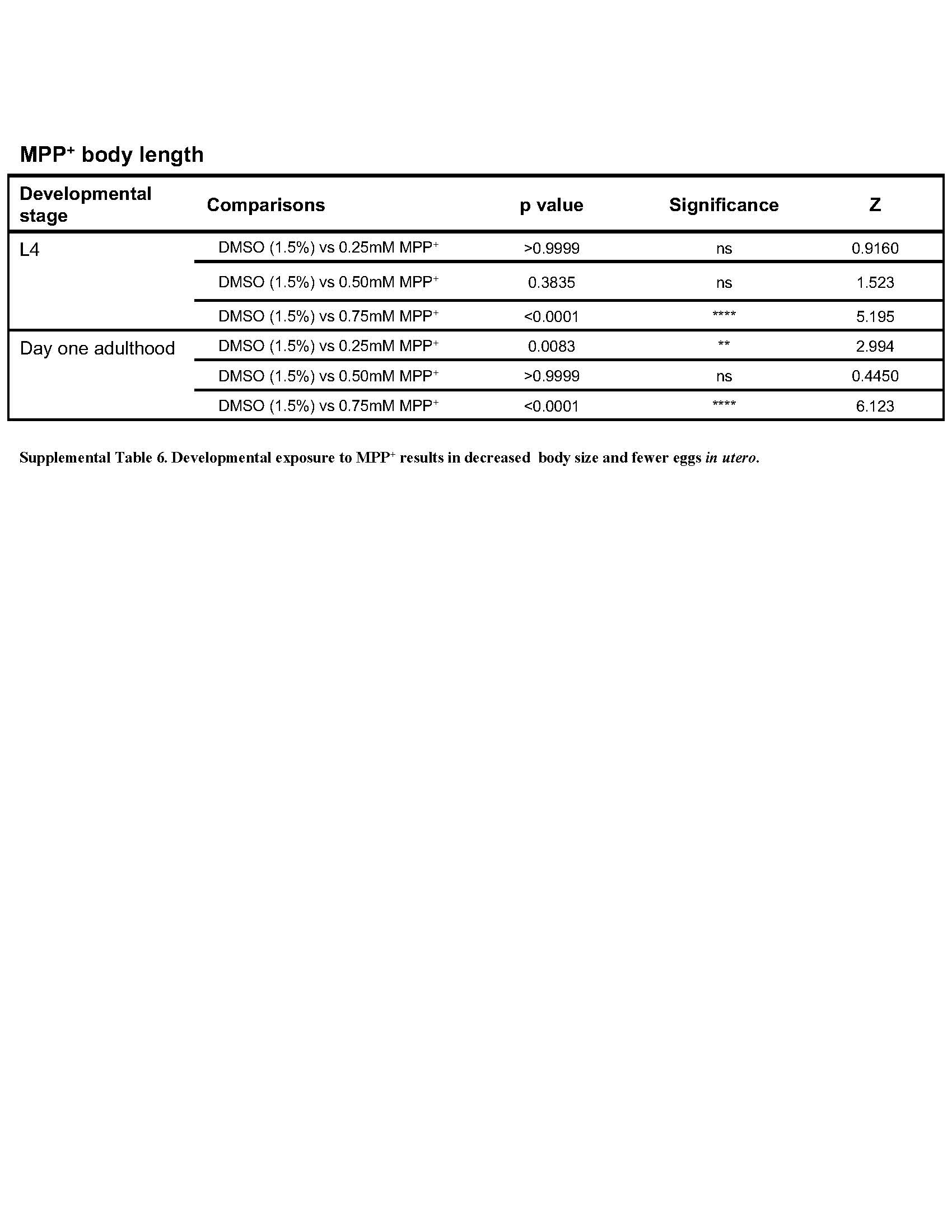

Supplement: Supplementary file 4 [file Image7.JPEG]

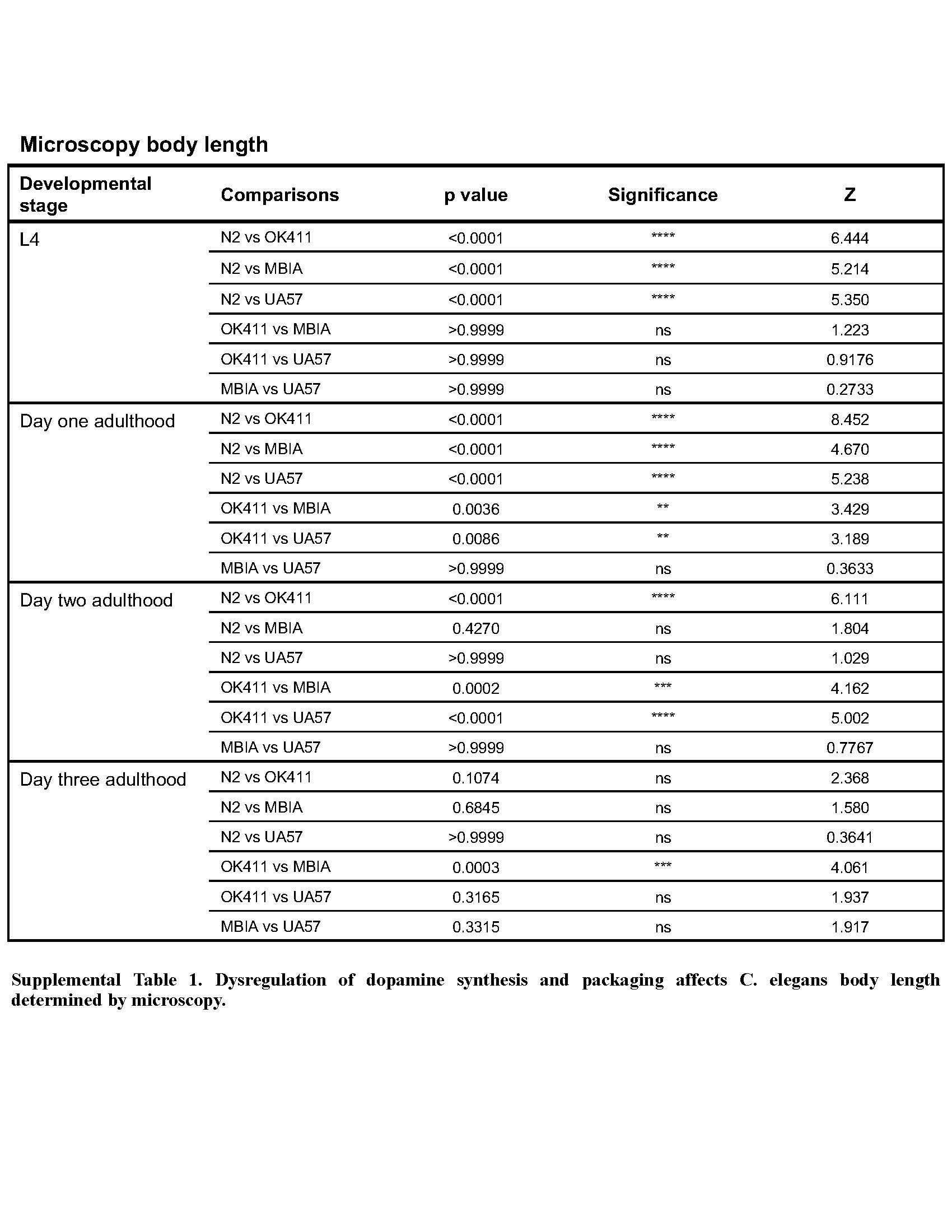

Supplement: Supplementary file 5 [file Image2.JPEG]

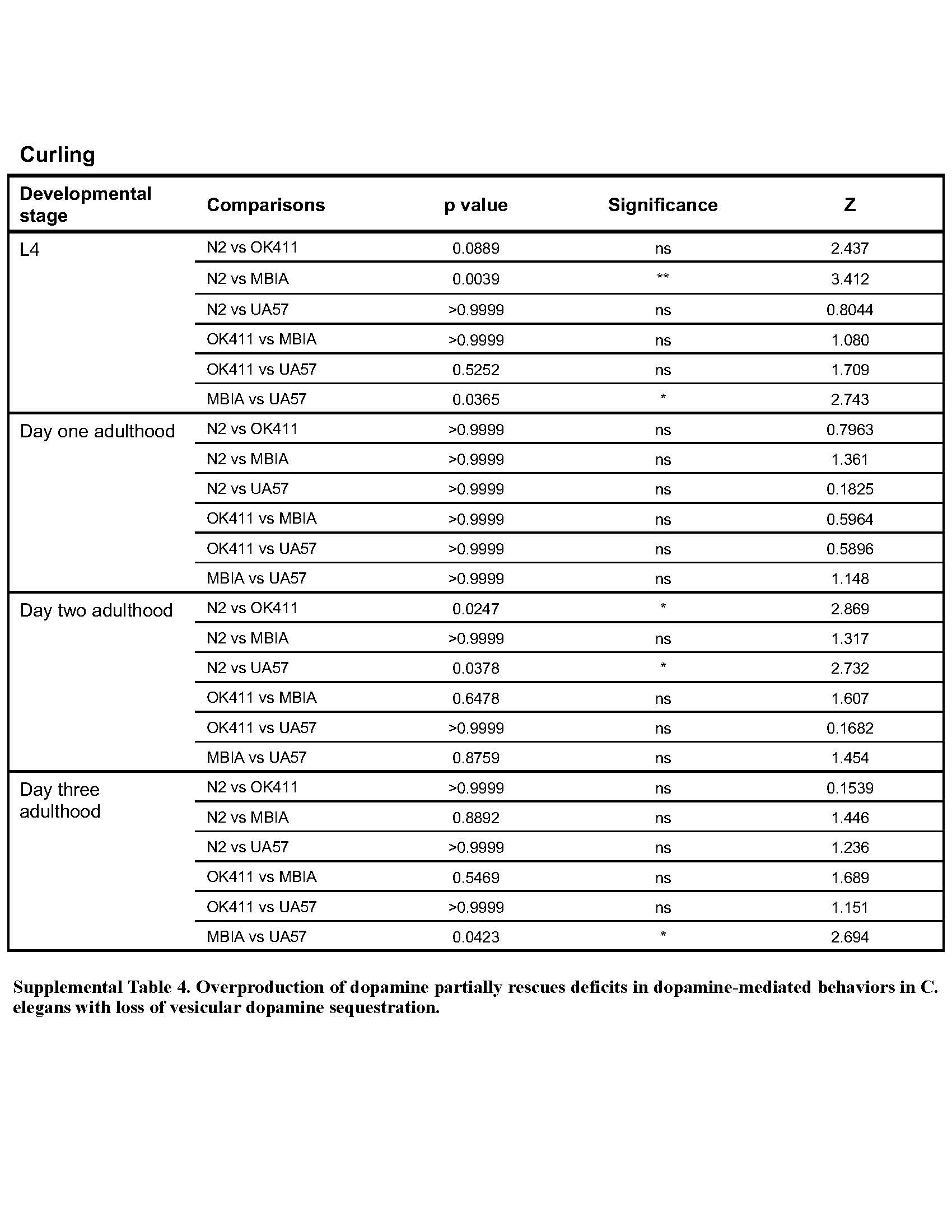

Supplement: Supplementary file 6 [file Image5.JPEG]

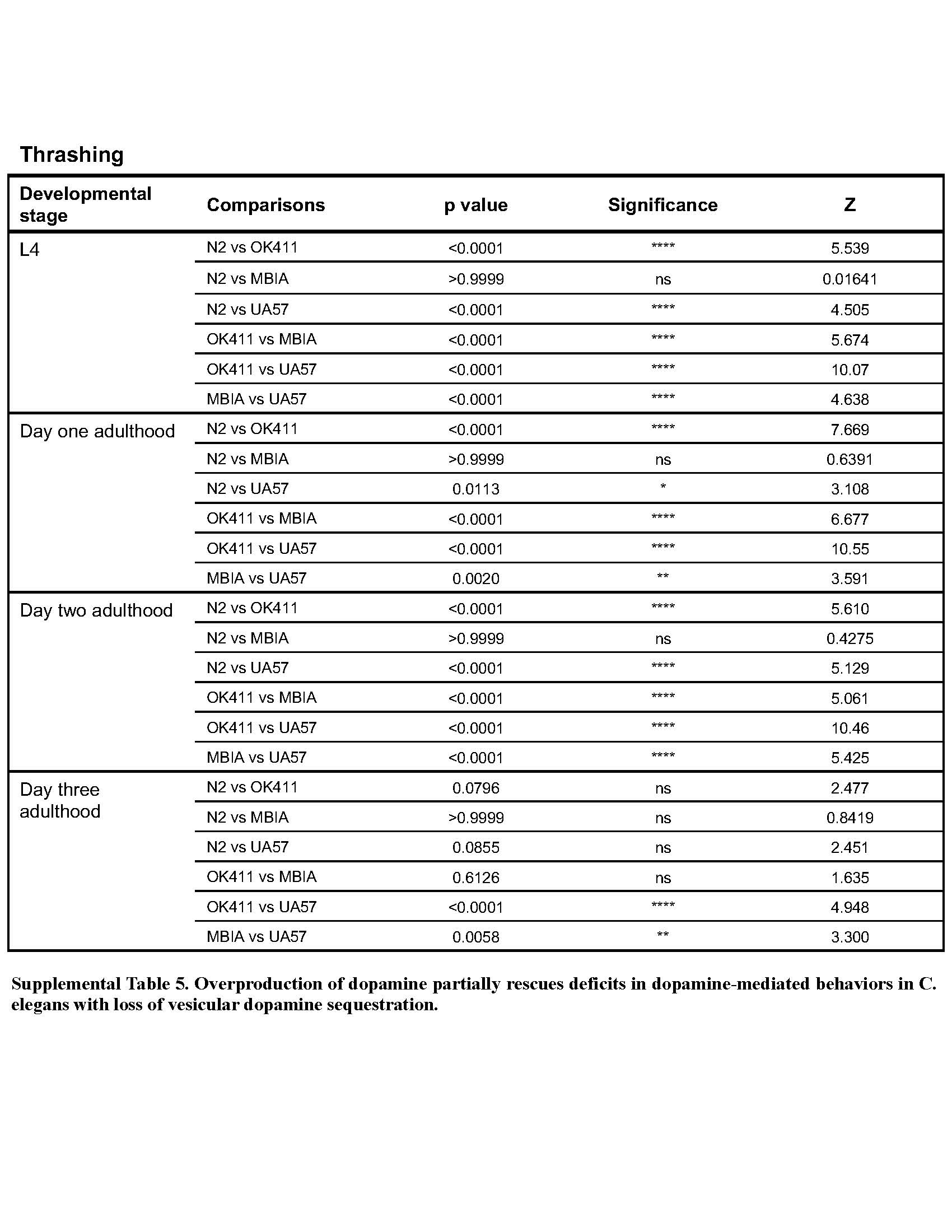

Supplement: Supplementary file 8 [file Image6.JPEG]
