# Supplementary material for: Effect of altered production and storage of dopamine on development and behavior in C. elegans
Source: Front Toxicol. 2024 Aug 16;6:1374866. doi: 10.3389/ftox.2024.1374866 (PMC11363549; doi:10.3389/ftox.2024.1374866)
Supplement: Supplementary file 7 [file DataSheet1.pdf]

Supplementary Material

Methods

MPP$^+$ exposure:

For 1-methyl-4-phenylpyridinium (MPP$^+$) treatment, agar (700µL normal growth medium) was aliquoted into individual wells of a 24-well plate. Once the agar set, live OP50 was applied for food source. Each well was treated with control (DMSO) or MPP$^+$ (50mM stock solution dissolved in DMSO diluted in M9) for a final concentration of 0.5% DMSO (equivalent to 0.25mM MPP$^+$), 1.5% DMSO (equivalent to 0.75mM MPP$^+$), 0.25mM MPP$^+$, 0.5mM MPP$^+$, and 0.75mM MPP$^+$. In addition, to determine whether DMSO influenced the measured outcomes, "non-treated" wells received an equal volume of M9. Each 24-well plate consisted of four replicates of each of the six treatments, and the experiment was repeated across four separate 24-well plates from unique synchronized populations of *C. elegans*. Treatments were applied to agar wells around the edge of the agar, avoiding the OP50 lawn, and allowed to absorb for 1h uncovered in a laminar flow hood before *C. elegans* were added. Approximately 30-50 L1 wild-type (N2) *C. elegans* were added to each well. For L4 analysis, worms were collected 48 hours after L1 plating. Day One Adult worms were analyzed 72 hours after L1 plating.
